# The Shelf Life of Milk—A Novel Concept for the Identification of Marker Peptides Using Multivariate Analysis

**DOI:** 10.3390/foods13060831

**Published:** 2024-03-08

**Authors:** Lisa-Carina Class, Gesine Kuhnen, Kim Lara Hanisch, Svenja Badekow, Sascha Rohn, Jürgen Kuballa

**Affiliations:** 1 GALAB Laboratories GmbH, Am Schleusengraben 7, 21029 Hamburg, Germany; lisa-carina.class@galab.de (L.-C.C.); gesine.kuhnen@galab.de (G.K.);; 2 Hamburg School of Food Science, Institute of Food Chemistry, University of Hamburg, Grindelallee 117, 20146 Hamburg, Germany; 3 Department of Food Chemistry and Analysis, Institute of Food Technology and Food Chemistry, Technische Universität Berlin, Gustav-Meyer-Allee 25, 13355 Berlin, Germany; rohn@tu-berlin.de

**Keywords:** fresh milk, shelf life, proteomic approach, marker peptides, UPLC-IMS-QToF, sensory analysis, multivariate statistics

## Abstract

The quality of food is influenced by several factors during production and storage. When using marker compounds, different steps in the production chain, as well as during storage, can be monitored. This might enable an optimum prediction of food’s shelf life and avoid food waste. Especially, proteoforms and peptides thereof can serve as indicators for exogenous influences. The development of a proteomics-based workflow for detecting and identifying differences in the proteome is complex and time-consuming. The aim of the study was to develop a fast and universal workflow with ultra-high temperature (UHT) milk as a proteinaceous model food with expectable changes in protein/peptide composition. To find an optimum shelf life without sticking to a theoretically fixed best-before date, new evaluation and analytical methods are needed. Consequently, a modeling approach was used to monitor the shelf life of the milk after it was treated thermally and stored. The different peptide profiles determined with high-resolution mass spectrometry (HRMS) showed a significant difference depending on the preparation method of the samples. Potential marker peptides were determined using orthogonal projections to latent structures discriminant analysis (OPLSDA) and principal component analysis (PCA) following a typical proteomics protocol with tryptic hydrolysis. An additional Python-based algorithm enabled the identification of eight potential tryptic marker peptides (with mass spectrometric structural indications *m*/*z* 885.4843, *m*/*z* 639.3500, *m*/*z* 635.8622, *m*/*z* 634.3570, *m*/*z* 412.7191, *m*/*z* 623.2967, *m*/*z* 880.4767, and *m*/*z* 692.4041), indicating the effect of the heat treatment. The developed workflow is flexible and can be easily adapted to different research questions in the field of peptide analysis. In particular, the process of feature identification can be carried out with significantly less effort than with conventional methods.

## 1. Introduction

Guaranteeing food safety and quality while saving resources is an issue of great concern for today’s (bio)economy [1]. Consumer trust in the quality and safety of food has been steadily damaged by several food scandals in recent years [2,3]. As a result, food safety issues are frequently a reason for excessive food waste [4]. Thus, it is important to continue the development of methods that simultaneously guarantee the safety of foods and minimize food waste. One way of minimizing food waste is to find process markers that can be used to monitor food quality during the food production process, enabling optimal use and “more exact” best-before dates [5].

In this context, milk is an important food product, as milk is wasted throughout the entire production chain. Microbiological risks and potential spoilage with subsequent sensorial decline can leave consumers feeling insecure [6,7]. Further, before milk is marketed, it undergoes a series of processing steps that influence its composition and stability. For example, these steps serve to control the fat content and free it from pathogenic microorganisms, thus preserving it. Traditionally, heat treatments are applied. To obtain a ‘sterile’ product, in which adverse microorganisms do not grow (or only at a much slower rate) during storage, ultrahigh temperature (UHT) treatments are used [8,9], enabling a relatively long shelf life. UHT milk treatments can be carried out using two different methods. Direct heating uses a steam injection method in which the milk is heated to 140–145 °C for 2–4 s [8,10,11]. Indirect heating can be carried out using a metal tube or metal plates. Heating is then carried out to 136–138 °C for 5–8 s [8,10,11].

However, shelf life (labeled on a product) is quite theoretical (and stipulated by legislation). It is the responsibility of the manufacturer to set a best-before date, but it is not specified in which way such an evaluation must be carried out. The manufacturer may define the conditions under which the best-before date is fixed [12]. Mostly, it is determined on the basis of microbiological and sensory methods [13], but beyond that, an individual safety margin is included. It is important to note that the “real” spoilage of food cannot be read from the best-before date due to the safety margin [12]. Therefore, a problem arises in that milk does not automatically spoil immediately after the best-before date expires. Moreover, it can usually fulfill the quality requirements for some time after this date [14]. This could be one of the reasons why food waste in households is gradually increasing [15]. Unfortunately, consumers rate the quality of products that are about to expire less favorably [16,17,18,19,20]. In this context, the shelf life date labeled on the food leads to misunderstandings, as consumers are more likely to throw away food that has passed its best-before date, even if this does not mean that the food is no longer safe [21,22,23,24]. According to the studies described by Zielińska et al., the microbiological safety of pasteurized milk is not at risk even six months after the best-before date has expired [25].

Therefore, milk might serve as a model for determining the (‘exact’) shelf life of foods, using a different method that better reflects the shelf life than the microbial or sensory approach. Due to the comprehensive data available on milk stability, the validity of a new method can be reviewed in comparison to the existing methods. A suitable approach to determine the shelf life of milk is to analyze the proteome [26]. Milk components, in particular milk proteins, offer a wide selection of nutritional, functional, and biological activities [26]. Changes in the proteome, as well as their interactions, are significantly responsible for the quality of the product [26,27,28,29]. Consequently, the proteome can serve as an indicator of environmental influences, processing such as heat treatments, or changes that occur during storage [30].

For some dairy products, it has already been shown that it can be beneficial to use peptides as marker substances [10,31,32,33,34]. According to von Oesen et al., selected (tryptic) peptides can be used as marker molecules to determine, for example, the content of whey protein in edam-type cheeses [34].

Traditionally, a bottom–up proteomics workflow is used for analyzing peptides. This means that proteolytic hydrolysis is carried out, starting from the (native) protein prior to characterization using mass spectrometry. The bottom–up workflow is considered to be a robust approach, and beyond that, it enables high-throughput analysis used for the identification and quantification of proteins in complex matrices [35]. Peptide profiles, ideally selected peptides, are suitable marker compounds, as they are mostly unique to their protein source [34]. The field of proteomics is well developed and can already answer a large number of research questions, but a chance to efficiently identify potential marker candidates is still needed.

Currently, the identification of marker peptides is still a major challenge as there are many different types of peptides and manual searches in databases are very time-consuming. Another challenge is that the successful identification of unique peptides depends on a large number of device-specific software or databases whose structures are not fully comprehensible to users. In addition, there are many post-translational modifications that can be very individual, possibly unique markers, but they depend on many influence factors and are only rarely considered in existing software.

The development of a workflow in combination with a bioinformatic evaluation of mass spectrometric data could be a promising step toward a reproducible basic workflow for future studies, which can be applied efficiently and with only minor adjustments to a variety of questions for the determination of the shelf life of food [12].

The aim of this study was to develop a workflow for the characterization of the milk proteome and determine the shelf life of milk based on the identification of potential (tryptic) marker peptides in order to reduce food waste. The approach was based on the hypothesis that the influence of processing can be determined by marker peptides of the milk proteome. The second hypothesis was that a workflow with a bottom–up approach in combination with computational analysis could enable the identification of marker compounds. To confirm these hypotheses, a workflow that detects these differences in the proteome and identifies features was developed. Furthermore, a study design was set up in which a change in the proteome of the milk was induced by exposure to heat. A bottom–up proteomics workflow was adapted and used as the analysis method. For enzymatic hydrolysis, trypsin, which is usually used in protein analytical procedures, was applied due to its availability and ease of use [36]. Milk proteins were the target of the analysis and, in this process, they were hydrolyzed via tryptic peptides. By targeting the milk proteins, standardization and improved reproducibility were the aims of this study. Moreover, the bottom–up approach with trypsin ensures adaptivity to future studies with other matrices. The mass spectrometric non-targeted measurement of the data was performed with liquid chromatograph–-electrospray ionization–ion mobility spectrometry–quadrupole time of flight (LC–ESI–IMS–QToF) technology. Initially, the workflow was developed using a model system consisting of pure standard milk proteins (caseins and whey proteins) to avoid matrix effects. Afterward, the workflow was applied to milk samples and compared to the protein model system. To identify differences, both heat-treated and untreated milk were used as sample materials. Due to the influence of external factors on the sensory profile of the milk, sensory analysis of the differently processed milk was also carried out. This made it possible to compare the sensory data with the analytically measured data. The mass spectrometric data were analyzed using statistical tools to detect trends, identify relationships between the individual data, and draw the corresponding conclusions [37]. Tryptic marker peptides were identified using computational data analysis due to the simple application and flexible adaptation to various issues and the different associated different methods. The algorithm performs a data comparison of previously theoretically generated possible tryptic peptides and their modifications with the measured real data, making the identification of markers significantly faster and more convenient compared to previous manual searches across various databases.

## 2. Materials and Methods

### 2.1. Reagents

HPLC-grade water and HPLC-grade acetonitrile were purchased from VWR International GmbH (Darmstadt, Germany). Formic acid was obtained from Biosolve B.V. (Valkenswaard, The Netherlands), while dithiothreitol (DTT), acetic acid, iodoacetamide (IAA), urea, and sodium bicarbonate were purchased from Sigma-Aldrich Chemie GmbH (Schnelldorf, Germany). Ammonium bicarbonate was obtained from Thermo Fisher Scientific Inc. (Waltham, MA, USA). Lock mass leucine enkephalin and the calibration standard for LC-IMS-QToF analysis were obtained from Waters Corp. (Milford, MA, USA).

### 2.2. Proteins

As model proteins, the six most abundant proteins in milk were chosen. α-Lactalbumin (≥90% purity) was purchased from US Biological Inc. (Salem, MA, USA). All other proteins, comprising β-lactoglobulin (≥90% purity), bovine serum albumin (BSA) (≥98% purity), α-casein (≥70% purity), β-casein (≥98% purity), and κ-casein (≥70% purity) were obtained from Sigma-Aldrich Chemie GmbH (Schnelldorf, Germany). Trypsin (from porcine pancreas, with a specific activity of 5000 usp-u/mg protein) was purchased from Carl Roth GmbH & Co. KG (Karlsruhe, Germany).

### 2.3. Sample Material

All milk samples were purchased from local supermarkets. The original milk sample was an organic ultra heat-treated (UHT) pasteurized milk with a fat content of 1.5% from a regional dairy farm (“Gläserne Molkerei”, Dechow, Germany). Steam injection was used to preserve the milk and remove pathogenic microorganisms. Therefore, the milk was heated to 142 °C for 4 s and homogenized at 200 bar.

### 2.4. Sample Preparation and Extraction

#### 2.4.1. Sample Preparation for the Model System

To achieve an impression of the impact of heat on the main milk proteins, the pure proteins (α-lactalbumin, β-lactoglobulin, BSA, α-casein, β-casein, and κ-casein) were initially used to avoid the influence of matrix effects. Prior to the heating process, the standard milk proteins were diluted according to the method used by Morschheuser et al. [38], while whey proteins (α-lactalbumin, β-lactoglobulin, BSA) were diluted in water (protein concentration: 4 mg/mL) and caseins were dissolved in sodium bicarbonate (protein concentration: 4 mg/mL) due to differences in their solubility [39,40]. Two different approaches for the following preparation were pursued: the individual protein model (model system 1) included half of the protein samples, which contained two samples of each protein solution. The protein solutions of model system 1 were enzymatically hydrolyzed directly after the proteins dissolved. Model system 2 included the other half of the samples. In this system, 0.25 mL of each protein solution was heated to 90 °C for 10 min before conducting enzymatic hydrolysis.

#### 2.4.2. Sample Preparation ‘Milk’

One part of the milk sample (500 mL) was heated to 90 °C for 10 min. For comparison, another part of the milk sample (500 mL) was used directly from the milk batch without any treatment. The milk was diluted according to Giansanti et al. so that the total protein content per individual sample was approx. 90 µg [36].

#### 2.4.3. Enzymatic Hydrolysis

Hydrolysis of the samples was performed with the serin protease trypsin, according to the method used by Giansanti et al. [36]. Briefly, milk samples and model systems 1 and 2 were concentrated until dryness and redissolved in 2 M of urea in water. Hydrolysis was carried out with an incubation time of 12 h at 37 °C. The last step of sample preparation was purification via solid phase extraction (SPE) with Sep-Pak^®^ C18 cartridges (Waters GmbH, Eschborn, Germany) [36]. Therefore, the cartridges were conditioned with 100% (*v*/*v*) acetonitrile, followed by an equilibration step with 0.6% (*v*/*v*) acetic acid in water. After applying the sample solutions, another washing step was carried out with 0.6% acetic acid solution, followed by elution with an aqueous 80% acetonitrile (0.6% acetic acid) solution. The tryptic peptide solutions were prepared for the mass spectrometric measurement by concentrating them to dryness and redissolving in 500 µL of 0.1% formic acid in water. Four replicates for each sample (heated and non-heated) preparation were prepared and analyzed, and duplicates for the standard protein solutions of the model system were also prepared.

### 2.5. Sensory Analysis

For the analysis of the sensory differences between heated and untreated milk, a simple descriptive test, according to the German official methodology described in §64 LFGB (German Food and Feed Code) L00.90–6:2015-06 [41], based on the German Institute for Standardization (DIN) norm 10964:2014-11 (BVL, 2015) [42], was used. A panel of eight trained persons aged from 20 to 45 were asked to test the milk samples. For a simple descriptive test, after giving written consent to participate, the participants received one sample of heated milk and another sample directly out of the milk package (untreated).

The procedure for the triangle test was different compared to the simple descriptive test. Three samples were submitted to the panel, only one of which was the heat-treated milk sample, the other two being the same. The triangle test was also performed according to the German official methodology according to §64 LFGB (German Food and Feed Code) L00.90–7:2021-11 [43] based on DIN EN ISO 4120 2021-06 [44].

### 2.6. UPLC-IMS-QToF Analysis

Mass spectrometric analysis of the samples was performed with an Acquity I-Class UPLC (ultrahigh performance liquid chromatography) system coupled with an ion mobility spectrometry quadrupole-time-of-flight mass spectrometer (a Vion IMS-QToF-MS) provided with an electrospray ionization source (ESI) (all Waters Corp., Milford, MA, USA). IMS allows for the measurement of the collision cross-section (CCS) [45,46]. With IMS measurements, the drift time can be obtained, enabling the calculation of CCS values [46]. CCS values offer information about the shape and size of an ion in the gas phase [45,46]. IMS was applied to improve the separation. For liquid chromatographic separation, an Acquity UPLC BEH C8 column (130 Å, 1.7 µm, 2.1 mm × 150 mm) (Waters Corp., Milford, MA, USA) was used. The column temperature was set to 40 °C, while the flow rate was 0.2 mL/min. Mobile phase A consisted of water with 0.1% formic acid (*v*/*v*), and mobile phase B was acetonitrile with 0.1% formic acid (*v*/*v*). The following chromatography gradient settings were chosen: 0.0 min (1% B), 1.0 min (1% B), 10.0 min (42% B), 12.0 min (85% B), 15.0 min (85% B), 16.5 min (1% B), and 19.5 min (1% B). The injection volume was 2 µL. Each sample was injected once. The samples were tempered at 10 °C in the autosampler. The positive ion mode was used for the detection, and the mass range was set to *m*/*z* 50–2000. Further parameters were set as follows: source temperature: 120 °C; desolvation temperature: 450 °C; cone gas flow: 50 L/h (nitrogen); desolvation gas flow: 800 L/h (nitrogen); capillary voltage: 0.50 kV; sample cone voltage: 40 V; and source offset voltage: 80 V. The HDMS^E^ (high-definition MS^E^) acquisition mode was used. With these parameters, the device simultaneously generates high- and low-energy spectra with a scan time of 0.150 s. The low-energy measurement was obtained using 4 V as the collision energy. Compared to the low-energy analysis, the high-energy measurement was performed by using a ramp with an elevated collision energy starting at 15 V and ending at 45 V. Nitrogen was used as the drift gas. The following parameters were used: IMS wave velocity of 300 m/s, IMS gas: 25 m/L, and IMS pulse height of 15.0 V. Lock mass correction was performed with leucine enkephalin (*m*/*z* 556.2766) every 2.5 min. Unifi 1.9.4.0 (Waters Corp., Milford, MA, USA) software was used to control the system and acquire the data.

### 2.7. Data Analysis and Statistics

The high-resolution mass spectrometric datasets were evaluated with Progenesis QI 2.3 software (Nonlinear Dynamics Ltd., Newcastle upon Tyne, UK). Mass correction, peak picking, deconvolution, retention time alignment, normalization, and multivariate analysis were accomplished. Principal component analysis (PCA) was performed as a form of multivariate analysis for analyzing the variance between the two different sample sets (heated vs. non-heated). With one-way analysis of variance (ANOVA) (*p*-value ≤ 0.05) and orthogonal projections to latent structures discriminant analysis (OPLS-DA), a massive reduction in detected features to a small number of statistically significant features was achieved. OPLS-DA was conducted with EZinfo 3.0.0.0. (Umetrics AB, Umeå, Sweden). In addition, hierarchical cluster analysis (HCA) was performed to assess the similarities and differences between the milk samples. This is a clustering method for comparing similarity patterns [47,48]. The distances between the samples were measured, and the results were displayed in the form of a dendrogram [47,48]. The same software was used to create the HCA as was used to create the PCA: Progenesis QI 2.3 (Nonlinear Dynamics Ltd., Newcastle upon Tyne, UK). The intensities of the features that are discussed are absolute intensities.

### 2.8. Feature Identification

**Figure 1:**
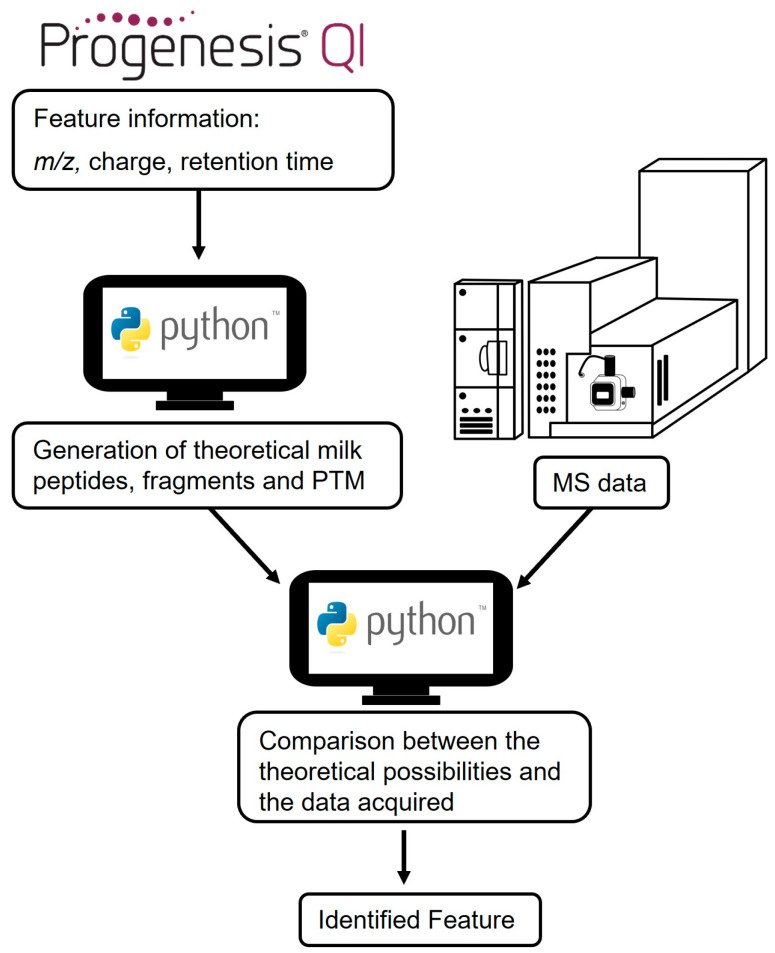
Schematic structure of the feature recognition workflow. The metadata (charge, *m*/*z*, and retention time) from the table of significant features generated in Progenesis QI were further processed with Python. Theoretically possible tryptic peptides, fragments, and post-translational modification (PTM) were generated and compared with the data obtained from mass spectrometry analysis, leading to the identified feature.

To identify the acquired mass spectrometric data using computational data analysis, the dataset had to be converted into a compatible data format. The datasets were converted from the device-specific uep-files to mzML-files with MSConvert (ProteoWizard, Version 3.0.20340). The feature identification algorithm was developed in Python (Version 3.9.16) and was divided into two parts. The first part is a filter for the theoretical possible tryptic peptides based on the *m*/*z* and the charge. Therefore, the protein sequences of the different milk proteins were obtained as fasta-files from uniprot.org (accessed 15 August 2023). The following milk proteins were considered: α-lactalbumin, β-lactoglobulin, BSA, α-s1-casein, α-s2-casein, β-casein, and κ-casein. The information on the features was obtained from the software Progenesis QI 2.3 (Nonlinear Dynamics Ltd., Newcastle upon Tyne, UK). The filter also considers modifications of the tryptic peptides as mass shifts. In the second part of the algorithm, a comparison between the theoretically possible tryptic peptides and the acquired mass spectrometric data takes place, which leads to the identified molecule. The schematic workflow is presented in Figure 1.

The packages applied in the algorithm were pandas (data structures for data analysis), numpy (scientific array computing), matplotlib (data plotting and visualization), and pyOpenMS. The latter is an open-source Python-based interface library for MS-based proteomics analysis that accesses OpenMS [49]. The statistically significant features were compared with the theoretically possible peptides that can be formed via tryptic hydrolysis of the milk proteins. The in silico algorithm in the first part of the workflow generates the theoretical masses and considers up to two missed cleavages of tryptic hydrolysis. Additionally, the first part of the workflow considers the following molecules for the calculation of the theoretical masses: theoretical tryptic peptides from the main proteins of the milk proteome (α-lactalbumin, β-lactoglobulin, BSA, α-s1-casein, α-s2-casein, β-casein, and κ-casein) as well as the b- and y-fragments of the tryptic peptides. Furthermore, post-translational modifications (methylation, oxidation, acetylation, lactulosyllysine, and Amadori products with galactose) were included in the second part of the workflow. The tolerance between the theoretical mass and the feature was 0.001%.

## 3. Results and Discussion

### 3.1. Sensory Analysis

Sensory analysis plays a major role in determining food alterations. When a change in flavor is perceptible, it can not only reduce consumer acceptance but also indicate spoilage of the food, which can either be less harmful or pose severe risks to human health. Sensory analysis is a widely used tool for determining the shelf life of milk as it reflects average consumer expectations and requirements [13,25,50,51]. In order to test whether consumers can perceive a sensory change between the differing samples of the present study, the heat-treated and non-treated milk samples were presented to a trained sensory panel for sensory analysis.

Two different sensory analyses were applied to describe the sensory properties of the milk samples. First, a simple descriptive test was conducted: panelists received one heat-treated and one non-heated sample. These samples were assessed according to appearance, odor, taste, and consistency, with taste showing the clearest differences. As expected, the panelists were able to distinguish the heat-treated sample from the non-heated sample by 80%. The attributes used to describe the flavor were primarily sweet, aqueous, milky, mild, acidic, cooked, creamy, intense, and greasy.

When comparing both samples, it was noticeable that the described characteristics of the heat-treated samples showed attributes (sweet, cooked, intensive, greasy, and milky) that were identified by the panel as clearly different from the norm. The non-heated milk samples were predominantly described by the panel as mild, aqueous, plain, slightly sweet, and creamy. This result met the expectations, as fresh cow’s milk is described with these properties (mild and creamy with sweet notes) in the literature [52].

The sensory distinction between heat-treated and non-heated milk has already been described in the literature. The cooked flavor described in the present study is associated with the Maillard reaction, which takes place between reducing sugars like lactose and amino groups of (milk) proteins when the milk is heated [53,54,55]. Flavor components connected with the Maillard reaction comprise Strecker aldehydes, sulfur- and nitrogen-containing compounds, maltol, and diacetyl [53,54,55]. Heat denaturation causes the release of volatile sulfur compounds from the serum protein, mainly β-lactoglobulin, which releases reactive sulfides and contributes to the “cooked” flavor [53,56,57,58,59]. The panel particularly emphasized the extremely sweet taste of the heat-treated sample. In addition to the Maillard reaction, this could also be explained by the degradation of lactose [55,60,61]. The molecular subunits of lactose are glucose and galactose. These monosaccharides, especially galactose, are released from lactose during degradation and have a sweeter flavor than pure lactose [61,62,63]. Moreover, it is known that lactulose, an isomer of lactose, is formed during milk thermal processing, being 1.5 times sweeter than lactose [63,64,65,66]. This could be another explanation for the panel’s sweeter perception of the heated milk sample. Further possible components providing a sweet taste impression are maltol, 2-acetyl-1-pyrroline, furaneol, and sotolon [53,55,67,68].

The greasy attribute of the heat-treated milk can be explained by lipid degradation due to the β-oxidation of free fatty acids associated with methyl ketones [53].

To validate the result of the simple descriptive test, a triangle test was carried out. The samples were not analyzed directly for their properties but for a deviating sample of a sample set consisting of three samples. Therefore, three samples were provided to the panel. One of the three samples was the heat-treated milk, while the other two samples were non-heated ones. The treated milk could be distinguished from the non-heated product by the panel that included eight people (*n* = 8, x = 8).

The significance level of the triangle test was 0.001%. The significance level can be read in Table A1 of §64 LFGB (German Food and Feed Code) L00.90–7:2021-11 [43] based on DIN EN ISO 4120 2021-06 [44]. The values given in Table A1 are the minimum number of correct answers required for significance at the specified risk level α (“risk level α” describes the probability of error) for the corresponding number of test subjects [43]. The assumption of “no difference” must be rejected when the number of correct answers is greater than or equal to the value in Table A1 [43]. In the present study, eight people from a panel were asked to participate in the test, all of whom recognized the deviating sample so that the deviating sample (heated milk) could be clearly distinguished from the standard sample (unheated) by the panel with 99.9%.

### 3.2. Data Processing and Statistical Analysis

The study was initially carried out using a modeling approach with pure milk proteins to test whether heat treatments affected the proteome without the influence of a whole complex matrix. Whey proteins and caseins were used as the standard milk proteins of choice. In order to obtain an overview of the changes in the tryptic peptides, a library was created through the use of Unifi software (Version 1.9.4.0). This library contained all tryptic peptides that can theoretically be produced during the tryptic degradation of the respective proteins.

After the measurement with LC-IMS-QToF, the comparison of the tryptic peptides from the theoretical and the experimental MS approach revealed that some tryptic peptides can only be observed in one of the two treatments (heated vs. non-heated). It was particularly noticeable that some of the tryptic peptides only occurred in the heated samples (e.g., tryptic peptides KILDK and IIAEK; Tables S1 and S2) or only in the non-heated samples (e.g., tryptic peptides IDALNENK and VLVLDTYKK; Table S2). A rather small number of tryptic peptides occurring in both sample types were detected, with significant differences in signal intensities. The results of the measurement of hydrolyzed milk protein standards are summarized in Tables S1–S7 (Supporting Information).

**Figure 2:**
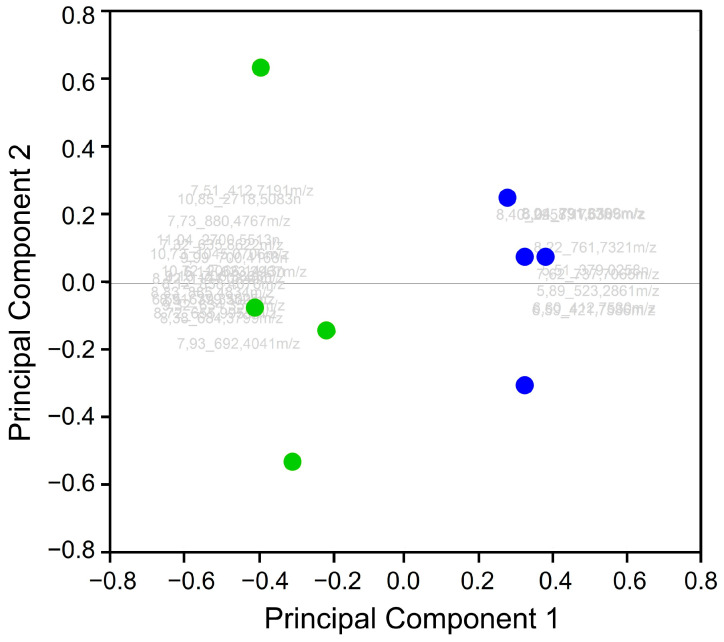
The two-dimensional principal component analysis of the milk samples. PCA scores plot in combination with the loadings of PC1 and PC2 of the direct enzymatic degraded milk samples: heated (green) and non-heated (blue) milk. The samples of these two groups are sample preparation replicates. PC1 and PC2 show 92.24% of the variance using 28 features. The assignment of the feature designation of the graphic is broken down in Table S8.

The uep-files from the mass spectrometric analysis of the milk samples were used for the statistical analysis with Progenesis QI software. Retention time alignment, peak detection, normalization, mass correction, and deconvolution of datasets were carried out separately for each measurement [69]. All replicates of the heated and non-heated samples were included in the PCA. The separation of the two groups in the PCA was obvious, even with no application of further filters, and the variance was 60.29% (PC1: 43.04% and PC2: 17.25%). To improve the variance and reduce the number of significant features, the statistical tool OPLS-DA and ANOVA evaluation were applied with a *p*-value of <0.05. The OPLS-DA showed that 31 features (=potential tryptic peptides) were significantly responsible for the variance of both sample groups (Figure S1). The PCA, which was performed with the features selected by the OPLS-DA, showed a variance of 90.12% (PC1: 82.08% and PC2: 8.04%). After that, ANOVA was applied to all of the features acquired. The ANOVA showed that 28 features fulfilled the requirements and showed significant variance in terms of distinguishing the different sample preparation methods. PCA applied with the ANOVA data had a variance of 92.24% (PC1: 86.81% and PC2: 5.43%). Figure 2 shows the PCA with the highest variance of 92.24%. The list of features that were extracted after applying the PCA (Figure 2) is summarized in Table 1.

**Figure 3:**
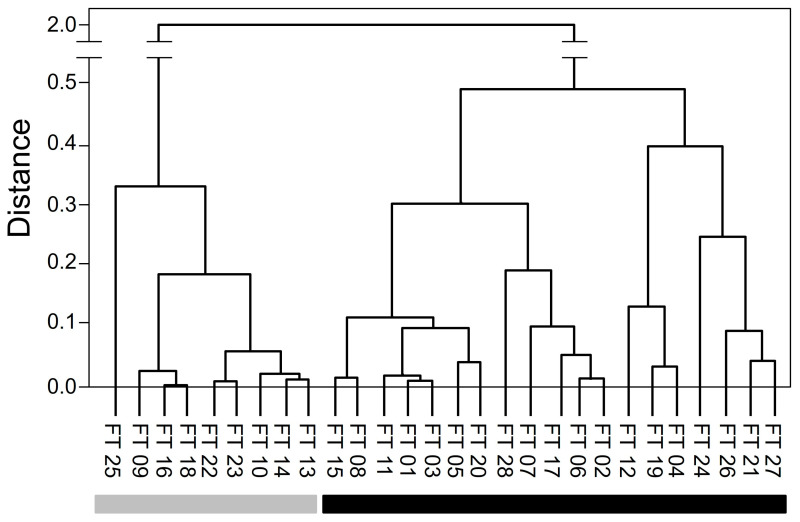
HCA dendrogram of the 28 significant features. The features marked in grey presented decreasing signal intensity due to the heat treatment. The features marked in black increased in signal intensity after heat treatment.

The HCA shown in Figure 3 displays the relationship between the 28 significant features of the two milk treatments in a hierarchical presentation. Obviously, the features are divided into two subgroups. The smaller the distance between the nodes of two characteristics, the more similar they were in terms of signal intensity. Nine features lost signal intensity when heated and are, therefore, similar. The other 19 features showed similarities due to the increased signal intensity after the heat treatment; these are also shown clustered in the dendrogram.

**Figure 4:**
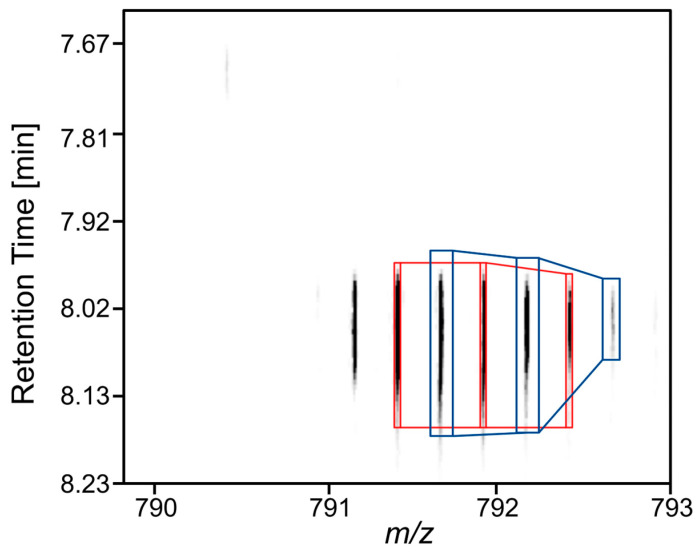
Ion map of feature FT 16 (*m*/*z* 791.6306) and FT 18 (*m*/*z* 791.3799). The ions marked in red are the three isotopes associated with feature FT 18 (*m*/*z* 791.3799), and the ions marked in blue are the isotopes that were assigned to FT 16 (*m*/*z* 791.6306). By observation of all the ions in the figure, it is visible that the ions have the same *m*/*z* distance from each other.

**Figure 5:**
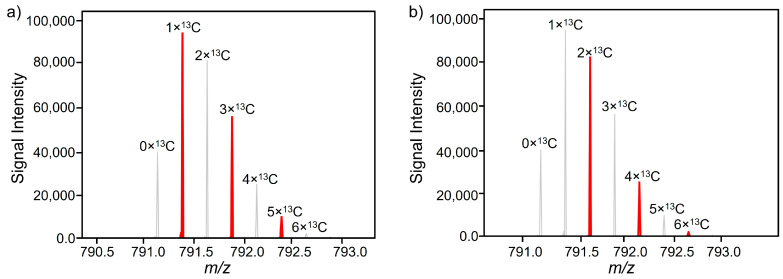
Ion spectra of feature FT 16 (*m*/*z* 791.6306) and FT 18 (*m*/*z* 791.3799). Feature FT 18 is shown in (**a**). The signals marked in red are the isotopes assigned by the software to FT 18. In (**b**), the red-marked peaks are associated with FT 16. The combination of both spectra shows that the signals labeled in red are ^13^C isotopes of the same molecule.

When the individual characteristics in the dendrogram (Figure 3) were analyzed in detail and compared with the data in Table 1, it was striking that features FT 23 and FT 22, FT 18 and FT 16, and features FT 08 and FT 15 were particularly similar. The data generated by Progenesis QI only showed an almost identical retention time for features FT 08 and FT 15. In the case of FT 18 and FT 16, the almost identical *m*/*z* and retention time were an unexpected result, as this indicated that both features originate from the same molecule. At first glance, these could be interpreted as isotopes. A more precise analysis of the features revealed that the Progenesis QI software did not determine the correct charge in this case. The software assumed that both features were assigned to a twofold charged molecule (Figure 4). However, when looking at the isotopic masses of both features, it was noticeable that the mass difference of the respective ions is about 0.25. This indicated that the two features (FT 16 and FT 18) are isotopes of the same fourfold charged molecule. This observation can also be derived from the ion map shown in Figure 4. It becomes more obvious in Figure 5, where the mass spectra of both features are presented. The peaks could be assigned to the parent ion with the smallest mass-to-charge ratio (*m*/*z* 791.1215), and the peaks with the increasing mass-to-charge ratio can be assigned to the following isotope variations: 1 × ^13^C (*m*/*z* 791.3799), 2 × ^13^C (*m*/*z* 791.6306), 3 × ^13^C (*m*/*z* 791.8753), 4 × ^13^C (*m*/*z* 792.1307), 5 × ^13^C (*m*/*z* 792.3818), and 6 × ^13^C-isotopes (*m*/*z* 792.6330). Thus, FT 16 was finally assigned to the 2 × ^13^C-based isotope and FT 18 to the 1 × ^13^C isotope. Therefore, both features belong to the same parent molecule. In the case of FT 23 and FT 22, the retention times were almost identical, and the neutral mass difference of 18 indicated a loss of water from the molecule.

### 3.3. Feature Identification

All 28 significant features generated by Progenesis QI after PCA, OPLS-DA, and ANOVA analysis were used for structural identification. The identification of the features was proceeded using Python. Therefore, algorithms were written to calculate the peptides produced from hydrolysis with trypsin. In addition, these theoretical tryptic peptides were compared with the experimental MS data to enable identification based on fragmentation patterns. In the first step, Python was used to calculate a selection of suitable tryptic milk peptides based on the *m*/*z* of the feature. This limits the range of tryptic peptides that can match the feature, leaving only a narrow selection of possibilities for further analysis. Additionally, not only unmodified tryptic peptides were considered, but possible modifications and fragments of tryptic peptides were also included. Furthermore, an assignment was made to the corresponding protein. The results obtained were the potential matches. In the next step, the measured data were analyzed for potential matches. The retention time of the individual features and the sequences of the theoretically possible tryptic peptide, as well as a possible modification, were used to analyze the acquired data. The isotopes and fragments found, such as the typical b- and y-fragments, indicated that the feature being searched for is a theoretically possible (modified) tryptic peptide. When a large number of y- and b- fragments is present [70], as expected, it could be assumed that the feature can be assigned to this molecule with the tryptic peptide being reconstructed almost exactly. The comparison was made with a relative tolerance of 0.001%.

In Table 2, the identified features are listed, and the corresponding results of the feature identification with Python have been provided in the Supplementary Materials (Tables S9–S28). Feature FT 03 was particularly striking because the threefold charged lactulosyllysine modification of the tryptic peptide with the single-letter code VLPVPQKAVPYPQR (β-casein) was assigned to this feature. This is a well-known modification in milk. Heat-treated milk usually undergoes the Maillard reaction [54]. The reactants are the sugar lactose and the amino acid lysine [54]. The Amadori product that is formed in milk is called lactulosyllysine [54].

**Figure 6:**
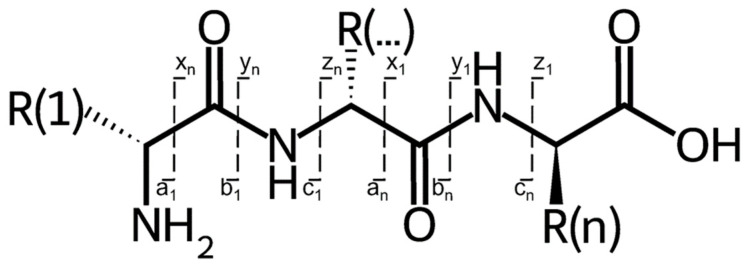
General fragmentation pattern of a peptide. The fragments are labeled x, y, and z from the C-terminal part of the peptide, and the a, b, and c fragments are labeled if the counting starts from the N-terminal part.

Furthermore, the features that were identified were mainly y-fragments and double-charged tryptic peptides. The y-fragments were, as according to Steen et al. [70,71] the predominant fragments when a quadrupole or a quadrupole-TOF was used as a mass analyzer. Figure 6 shows the generic structure of a peptide and the possible fragmentation types. The mentioned y-fragments were molecules that are formed when the nomenclature starts from the C-terminal backbone of the peptide.

The features of *m*/*z* 885.4834 (α-s2-casein), 635.8622 (β-casein), 412.7191 (α-s1-casein), and 692.4041 (β-casein) were identified as y-fragments of the respective tryptic peptide (Table 2). A more detailed analysis of the identified fragments showed that the two y-fragments assigned to β-casein (FT 04 and FT 28) originated from the same tryptic peptide (DMPIQAFLLYQEPVLGPVR). Feature FT 04 is the y11++ fragment, and the FT 28 feature is the y12++ fragment. Different fragments of this tryptic peptide were also identified in other studies, but the samples were not analyzed in terms of storage or process changes to bovine milk [72,73].

**Figure 7:**
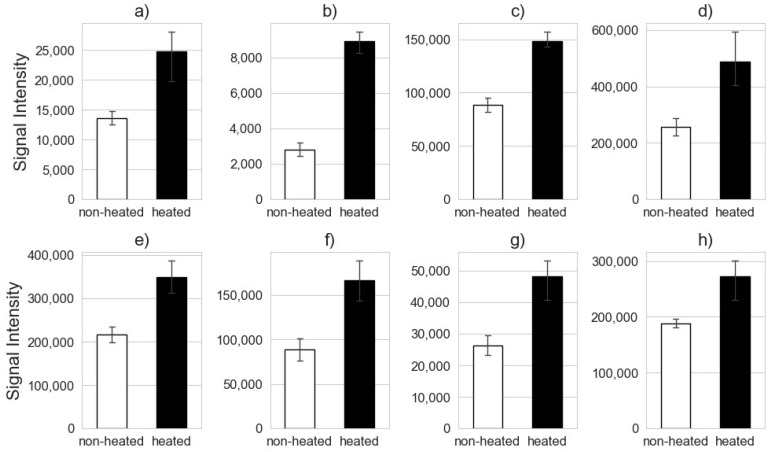
Signal intensities of the identified features depending on heat treatment. The mean value of the signal intensities from the low-energy spectra is shown, where (**a**) describes FT 02, (**b**) FT 03, (**c**) FT 04, (**d**) FT 05, (**e**) FT 12, (**f**) FT 24, (**g**) FT 26, and (**h**) FT 28.

In addition, the following double-charged features with the *m*/*z* 634.3570 (α-s1-casein), 623.2967 (β-lactoglobulin), and 880.4767 (α-s1-casein) were also identified (Table 2). A comparison of the signal intensities of the individual eight features showed that each one had a significantly higher signal intensity after heating than in the unheated state. Figure 7 shows the mean value of the signal intensities out of the low-energy spectrum. These eight features can also be compared with the results of the model approach (milk protein standards).

It seems obvious that the matrix had a major influence on the proteome in terms of heat-induced changes. In contrast to the results of the model approach, the results relating to the real milk showed that none of the significant features identified in the models occurred exclusively in one processing method (heated vs. non-heated). For example, FT 03, which is assigned to the lactulosyllysine modification of the tryptic peptide VLPVPQKAVPYPQR in β-casein, was only found in the heated samples in the model approach without lactulosyllysine modification (Table S2). Furthermore, the lactulosyllysine modification of the tryptic peptide exclusively occurred in the milk, as no lactose is present in the reaction solution of the model approach. The modified tryptic peptide (VLPVPQKAVPYPQR) was found in both treatments of the real food model; however, its signal intensity was higher in the heated samples (Figure 7). It should be noted that the modeling approach and the real food approach are only comparable to a limited extent, as different quantities of the sample materials were used for heating. This may have influenced the results.

The lactulosyllysine-modified feature (FT 03) is a product of the Maillard reaction, which is typically induced by heat. However, the short heating period applied can already induce the Maillard reaction [54,62]. Obviously, the samples with a higher signal intensity of FT 03 (Figure 7) were heated for 10 min at 90 °C in addition to the pasteurization step. Therefore, the progression of the Maillard reaction can be read from this feature. The observation that heating time and the temperature applied have roles in the formation of marker peptides is well known. Meltretter et al. published a study that investigated the change in the peptide profile during storage and thermal milk treatments. For their analysis, they used immobilized metal affinity chromatography coupled to matrix-assisted laser desorption/ionization time-of-flight [74]. In contrast to the study presented here, Meltretter et al. investigated peptides that are not formed via enzymatic hydrolysis but by the attack of radicals on the protein backbone as a result of the heat-induced Maillard reaction [74]. Moreover, the identification of the potential marker peptides in that study was performed by searching the SWISS-PROT database [74]. Generally, global or commercial databases have to be referenced first when it is not possible to realize how exactly database synchronization works. With a Python-based workflow, every step can be directly traced and modified when necessary.

Another example is feature FT 24, which was assigned to the tryptic peptide TPEVDDEALEK originating from β-lactoglobulin. This tryptic peptide was only found in the heated samples of the model approach (Table S6). Feature FT 24 was also found and identified in the milk samples, although in different concentrations (Table 2 and Figure 7). This is a phenomenon that is already described in the literature [34,75].

The limited comparability of proteins with or without food matrix was also shown in other studies. For example, von Oesen et al. conducted a study on different whey-containing cheese matrices [34]. Von Oesen et al. investigated whey protein content during cheese manufacturing (esp. whey protein-enriched cheese) and identified tryptic marker peptides for the quantification of whey protein content. That study highlighted the fact that matrix effects influence the analysis of the proteome, as the initial proteome itself changes does not change, but a certain degradation occurs, and different matrices might affect extraction strategies [34,75].

β-casein-derived tryptic peptide DMPIQAFLLYQEPVLGPVR, α-s1-casein-dervied peptide YLGYLEQLLR, α-s1-casein-derived peptide HQGLPQEVLNENLLR, and TPEVDDEALEK reflect the thermal treatment of milk and are also already known as tryptic marker peptides due to the results of other studies [76]. Van Vlierbergh et al. selected those four tryptic peptides as well as four further tryptic peptides as suitable tryptic marker peptides to monitor multiple and often-applied food processing techniques for milk [76]. The results for the β-lactoglobulin-derived tryptic peptide TPEVDDEALEK (FT 24) were particularly striking as the signal intensity of the tryptic peptide appeared to decrease as the degree of milk processing increased (comparison of freeze-dried raw milk and freeze-dried UHT milk) [76]. According to van Vlierbergh et al., the concentration of TPEVDDEALEK would be expected to decrease when the UHT milk is heated a second time, but the opposite was the case here. The present study showed that this peptide’s signal intensity in heated UHT milk increased compared to non-heated UHT milk. (Figure 7).

Dalabasmaz et al. (2017) characterized marker peptides in milk for differentiating UHT milk from more gently heated milk [77]. They considered peptides as potential markers due to the fact that they are naturally released during the pasteurization/heating process. However, those results were ambivalent as some peptides increased while others decreased in concentration [77]. The signal intensity of some of the potential marker peptides described by Dalabasmaz et al. (2017) increased the higher the sample was heated [77]. This is an observation that was also noted in the present study. However, it should be noted that the potential marker peptides in the study described by Dalabasmaz et al. (2017) differ from those identified as significant potential markers in the present study (Table 2). An explanation for the differences in the considered marker peptides may depend on the different analytical approaches used in the studies, especially in terms of sample preparation. In this study, enzymatic hydrolysis was carried out with trypsin, and the released peptides were subsequently analyzed using UPLC-IMS-ToF; however, the peptides described by Dalabasmaz et al. (2017) were of endogenous origin, released by enzymes of the original cheese milk and analyzed using MALDI-ToF-MS. For this reason, the results described by Dalabasmaz et al. (2017) are not exactly comparable with the results presented in this study [77]. A similarity of both studies is the marker peptides deriving from α-s1-casein. These peptides were present in features FT 05 (YLGYLEQLLR), FT 12 (EPMIGVNQELAYFYPELFR), and FT 26 (HQGLPQEVLNENLLR) in the present study.

In a later study, Dalabasmaz et al. (2019) described proteolysis during milk storage, with the marker peptides again being identified [10]. In that study, the milk was not heated to cause a change in the proteome; instead, it was stored until the best-before date was reached [10]. Enzymes active during storage, such as plasmin, were responsible for the release of peptides [10]. This distinguishes the research conducted by Dalabasmaz et al. (2019) from the study presented here. Nevertheless, similarities can be observed between the studies. It is striking that the marker peptides selected in the study described by Dalabasmaz et al. (2019) were mainly from β-casein, and the peptide levels increased significantly towards the end of the shelf life [10]. The indication that caseins reflect changes in external conditions is also evident in the studies presented here, as seven of the eight identified potential tryptic marker peptides are assigned to the casein fraction of the milk proteome (Table 2).

## 4. Conclusions

This study is primarily a method development study. Consequently, there is no plan to publish the data in a public database as would be common for a proteomic study. In the future, when different proteomic studies have been carried out based on the presented methodology, a workflow for the selection and identification of marker peptides will be developed. As expected, it was shown that the influence of external conditions, such as thermal processing, cannot only be perceived from a sensory perspective but also clearly determined from the proteome, making protein analysis an efficient tool for the determination of biomarkers/processing marker compounds. Furthermore, it can also yield unbiased information compared to sensory data resulting from human participants. Obviously, this discovery is not novel, but the study also emphasizes that the inclusion of bioinformatics when interpreting the results of the analysis can be beneficial. The presented workflow is flexible and can be easily adapted to various other research questions in food protein/peptide analysis. For other studies, sample preparation and the databases used in the identification process must be customized for the proteins/peptides of interest. As the presented study primarily described the development of a workflow, the number of samples was rather low; for further studies, the number of samples needs to be pre-evaluated with regard to final statistical evaluations.

Another limitation is that not all features were identified in terms of their exact chemical structure. This can be caused by two factors in the process. One is a limitation of the database. Only the most common modifications are considered in the identification process. More than that, the identification was limited to tryptic peptides without consideration of the enzymatic hydrolysis of enzymes that naturally occurs in milk when stored for a certain period. Considering all of the possible modifications and reactions is hardly achievable due to the diverse reactivities and potential follow-up degradations of some of the peptides.

The inclusion of bioinformatics tools offers a wide range of possibilities. Large datasets can be processed, and the analytical applications are diverse. The developed algorithm can identify characteristics/features with significantly less effort than before. There is no need to manually trawl through datasets; the script compares the dataset directly with theoretical possibilities. This minimizes susceptibility to errors and saves an enormous amount of work and time. The workflow developed shows that the interaction of device-specific software and the use of bioinformatics tools are not limiting each other but can be used together, thus favoring better analysis results.

## Figures and Tables

**Table 1:** The table shows the results of the feature metadata obtained from the PCA analysis: summary of the feature *m*/*z*, charge, retention time, CCS-value, ANOVA *p*-value, and q-value. The table includes all the information used for the PCA and the assignment of the significant features to differentiate the sample preparation methods.

Feature Name	*m*/*z*	Charge	Retention Time [min]	CCS [Å]	ANOVA (*p*)	q Value
FT 01	655.9955	3	8.72	592.71	7.98 × 10^−5^	4.79 × 10^−3^
FT 02	885.4834	2	8.83	454.25	1.87 × 10^−4^	6.62 × 10^−3^
FT 03	639.3500	3	6.55	526.31	1.89 × 10^−4^	6.62 × 10^−3^
FT 04	635.8622	2	7.32	376.85	2.39 × 10^−4^	6.87 × 10^−3^
FT 05	634.3570	2	9.32	391.93	5.33 × 10^−4^	8.19 × 10^−3^
FT 06	821.3864	1	8.91	281.99	6.24 × 10^−4^	8.44 × 10^−3^
FT 07	858.4076	2	8.12	438.96	1.15 × 10^−3^	1.11 × 10^−2^
FT 08	697.0494	3	10.72	539.40	1.76 × 10^−3^	1.23 × 10^−2^
FT 09	761.7321	3	8.22	559.93	2.01 × 10^−3^	1.26 × 10^−2^
FT 10	443.0417	1	5.51	191.23	2.03 × 10^−3^	1.26 × 10^−2^
FT 11	684.3799	3	8.38	539.73	2.56 × 10^−3^	1.32 × 10^−2^
FT 12	412.7191	2	7.51	319.19	2.81 × 10^−3^	1.32 × 10^−2^
FT 13	523.2861	2	5.89	349.85	3.26 × 10^−3^	1.36 × 10^−2^
FT 14	737.7065	3	7.62	560.52	3.38 × 10^−3^	1.37 × 10^−2^
FT 15	1045.0706	2	10.73	494.44	3.55 × 10^−3^	1.39 × 10^−2^
FT 16	791.6306	2	8.04	393.91	4.55 × 10^−3^	1.47 × 10^−2^
FT 17	748.3706	1	9.16	266.04	6.14 × 10^−3^	1.61 × 10^−2^
FT 18	791.3799	2	8.04	388.88	6.48 × 10^−3^	1.65 × 10^−2^
FT 19	742.4507	1	9.99	268.93	6.64 × 10^−3^	1.66 × 10^−2^
FT 20	1141.0840	2	8.42	520.30	7.52 × 10^−3^	1.77 × 10^−2^
FT 21	908.5183	3	11.04	601.58	9.02 × 10^−3^	1.93 × 10^−2^
FT 22	412.7530	2	6.60	319.19	1.35 × 10^−2^	2.31 × 10^−2^
FT 23	421.7586	2	6.59	323.67	1.45 × 10^−2^	2.41 × 10^−2^
FT 24	623.2967	2	6.11	377.12	1.67 × 10^−2^	2.58 × 10^−2^
FT 25	761.0597	3	8.40	567.39	2.82 × 10^−2^	3.46 × 10^−2^
FT 26	880.4767	2	7.73	459.62	3.39 × 10^−2^	3.82 × 10^−2^
FT 27	914.5040	3	10.85	601.45	3.84 × 10^−2^	4.08 × 10^−2^
FT 28	692.4041	2	7.93	390.73	4.74 × 10^−2^	4.67 × 10^−2^

**Table 2:** The results of the identified tryptic milk peptides are shown. Eight of the twenty-eight features that are significantly responsible for the variance of the samples were identified. In addition to feature designation, retention time, *m*/*z*, the charge, modification, fragment type, and assignment to the peptide sequence and corresponding protein are listed.

Feature Name	Retentiontime [min]	*m*/*z*	Charge	Modification Type	Fragment	Tryptic Peptide	Protein
FT 02	8.83	885.4834	2	-	y15++	FPQYLQYLYQGPIVLNPWDQVK	α-s2-casein
FT 03	6.55	639.3500	3	Lactulosyl-lysine	-	VLPVPQKAVPYPQR	β-casein
FT 04	7.32	635.8622	2	-	y11++	DMPIQAFLLYQEPVLGPVR	β-casein
FT 05	9.32	634.3570	2	-	-	YLGYLEQLLR	α-s1-casein
FT 12	7.51	412.7191	2	-	y6++	EPMIGVNQELAYFYPELFR	α-s1-casein
FT 24	6.11	623.2967	2	-	-	TPEVDDEALEK	β-lactoglobulin
FT 26	7.73	880.4767	2	-	-	HQGLPQEVLNENLLR	α-s1-casein
FT 28	7.93	692.4041	2	-	y12++	DMPIQAFLLYQEPVLGPVR	β-casein

## Data Availability

The original contributions presented in the study are included in the article, further inquiries can be directed to the corresponding author. The data are not publicly available due to them being contained within a very large dataset.

## Supplementary Materials

The following supporting information can be downloaded at: https://www.mdpi.com/article/10.3390/foods13060831/s1, Figure S1: Loading plot (S-plot) of the OPLS-DA to differentiate heated and non-heated milk samples; Table S1: Comparison of the tryptic peptide signal intensities obtained from α-Lactalbumin standard after tryptic digestion; Table S2: Comparison of the tryptic peptide signal intensities obtained from β-Lactoglobulin standard after tryptic digestion; Table S3: Comparison of the tryptic peptide signal intensities obtained from bovine serum albumin (BSA) standard after tryptic digestion; Table S4: Comparison of the tryptic peptide signal intensities obtained from α-s1-Casein standard after tryptic digestion; Table S5: Comparison of the tryptic peptide signal intensities obtained from α-s2-Casein standard after tryptic digestion; Table S6: Comparison of the tryptic peptide signal intensities obtained from β-Casein standard after tryptic digestion; Table S7: Comparison of the tryptic peptide signal intensities obtained from κ-Casein standard after tryptic digestion; Table S8: Definition of the feature names; Table S9: Comparison of the detected *m*/*z* from feature FT 02 and the assigned fragments of the tryptic peptide “FPQYLQYLYQGPIVLNPWDQVK”; Table S10: Comparison of the detected *m*/*z* from feature FT 02 and the assigned fragments of the tryptic peptide “FPQYLQYLYQGPIVLNPWDQVK”; Table S11: Comparison of the detected *m*/*z* from feature FT 03 and the assigned isotopes of the tryptic peptide “VLPVPQKAVPYPQR” modified with lactulosyllysine; Table S12: Comparison of the detected *m*/*z* from feature FT 03 and the assigned fragments of the tryptic peptide “VLPVPQKAVPYPQR” modified with lactulosyllysine; Table S13: Comparison of the detected *m*/*z* from feature FT 03 and the assigned fragments of the tryptic peptide “VLPVPQKAVPYPQR” modified with lactulosyllysine; Table S14: Comparison of the detected *m*/*z* from feature FT 04 and the assigned fragments of the tryptic peptide “DMPIQAFLLYQEPVLGPVR”; Table S15: Comparison of the detected m/z from feature FT 04 and the assigned fragments of the tryptic peptide “DMPIQAFLLYQEPVLGPVR”; Table S16: Comparison of the detected *m*/*z* from feature FT 05 and the assigned isotopes of the tryptic peptide “YLGYLEQLLR”; Table S17: Comparison of the detected *m*/*z* from feature FT 05 and the assigned fragments of the tryptic peptide “YLGYLEQLLR”; Table S18: Comparison of the detected *m*/*z* from feature FT 05 and the assigned fragments of the tryptic peptide “YLGYLEQLLR”; Table S19: Comparison of the detected *m*/*z* from feature FT 12 the assigned fragments of the tryptic peptide “EPMIGVNQELAYFYPELFR”; Table S20: Comparison of the detected *m*/*z* from feature FT 12 and the assigned fragments of the tryptic peptide “EPMIGVNQELAY-FYPELFR”; Table S21: Comparison of the detected *m*/*z* from feature FT 24 and the assigned isotopes of the tryptic peptide “TPEVDDEALEK”; Table S22: Comparison of the detected *m*/*z* from feature FT 24 and the assigned fragments of the tryptic peptide “TPEVDDEALEK”; Table S23: Comparison of the detected *m*/*z* from feature FT 24 and the assigned fragments of the tryptic peptide “TPEVDDEALEK”; Table S24: Comparison of the detected *m*/*z* from feature FT 26 and the assigned isotopes of the tryptic peptide “HQGLPQEVLNENLLR”; Table S25: Comparison of the detected *m*/*z* from feature FT 26 and the assigned fragments of the tryptic peptide “HQGLPQEVLNENLLR”; Table S26: Comparison of the detected *m*/*z* from feature FT 26 and the assigned fragments of the tryptic peptide “HQGLPQEVLNENLLR”; Table S27: Comparison of the detected *m*/*z* from feature FT 28 and the assigned fragments of the tryptic peptide “DMPIQAFLLYQEPVLGPVR”; Table S28: Comparison of the detected *m*/*z* from feature FT 28 and the assigned fragments of the tryptic peptide “DMPIQAFLLYQEPVLGPVR”.

## References

[1] Grunert K.G. (2005). Food quality and safety: Consumer perception and demand. Eur. Rev. Agric. Econ..

[2] Ling E.K. (2020). Integrity of food supply chain: Going beyond food safety and food quality Siti Norida Wahab. Int. J. Product. Qual. Manag..

[3] Wang J., Yue H. (2017). Food safety pre-warning system based on data mining for a sustainable food supply chain. Food Control.

[4] Toma L., Revoredo-Giha C., Costa-Font M., Thompson B. (2020). Food Waste and Food Safety Linkages along the Supply Chain. EuroChoices.

[5] Corradini M.G. (2018). Shelf Life of Food Products: From Open Labeling to Real-Time Measurements. Annu. Rev. Food Sci. Technol..

[6] Scherhaufer S., Moates G., Hartikainen H., Waldron K., Obersteiner G. (2018). Environmental impacts of food waste in Europe. Waste Manag..

[7] Gustavsson J., Cederberg C., Sonesson U., Emanuelsson A. (2013). The Methodology of the FAO Study: “Global Food Losses and Food Waste-Extent, Causes and Prevention”.

[8] Deeth H.C., Datta N. (2011). Heat Treatment of Milk: Ultra-High Temperature Treatment (UHT): Heating Systems. Encycl. Dairy Sci. Second Ed..

[9] Linares D.M., Del Río B., Ladero V., Martínez N., Fernández M., Martín M.C., Álvarez M.A. (2012). Factors influencing biogenic amines accumulation in dairy products. Front. Microbiol..

[10] Dalabasmaz S., Dittrich D., Kellner I., Drewello T., Pischetsrieder M. (2019). Identification of peptides reflecting the storage of UHT milk by MALDI-TOF-MS peptide profiling. J. Proteom..

[11] Belitz H.-D., Grosch W., Schieberle P. (2008). Lehrbuch der Lebensmittelchemie.

[12] Class L., Kuhnen G., Rohn S., Kuballa J. (2021). Diving Deep into the Data: A Review of Deep Learning Approaches and Potential Applications in Foodomics. Foods.

[13] Martin N.H., Ranieri M.L., Murphy S.C., Ralyea R.D., Wiedmann M., Boor K.J. (2011). Results from raw milk microbiological tests do not predict the shelf-life performance of commercially pasteurized fluid milk. J. Dairy Sci..

[14] Beckmann S., Scheller S. (2011). Bestimmung des Mindesthaltbarkeitsdatums. Der Leb.—Ernährung Aktuell.

[15] Kretschmer B., Smith C., Watkins E., Allen B., Buckwell A., Desbarats J., Kieve D. (2013). Technology Options for Feeding 10 Billion People—Recycling Agricultural, Forestry & Food Wastes and Residues for Sustainable Bioenergy and Biomaterials. Inst. Eur. Environ. Policy.

[16] Newsome R., Balestrini C.G., Baum M.D., Corby J., Fisher W., Goodburn K., Labuza T.P., Prince G., Thesmar H.S., Yiannas F. (2014). Applications and perceptions of date labeling of food. Compr. Rev. Food Sci. Food Saf..

[17] Wansink B., Wright A.O. (2006). “Best if used by...” How freshness dating influences food acceptance. J. Food Sci..

[18] Wilson N.L.W., Rickard B.J., Saputo R., Ho S.T. (2017). Food waste: The role of date labels, package size, and product category. Food Qual. Prefer..

[19] Theotokis A., Pramatari K., Tsiros M. (2012). Effects of Expiration Date-Based Pricing on Brand Image Perceptions. J. Retail..

[20] Buttlar B., Löwenstein L., Geske M.S., Ahlmer H., Walther E. (2021). Love food, hate waste? Ambivalence towards food Fosters people’s willingness to waste food. Sustainability.

[21] Kavanaugh M., Quinlan J.J. (2020). Consumer knowledge and behaviors regarding food date labels and food waste. Food Control.

[22] Patra D., Feng S., Howard J.W. (2022). Confusion of food-date label with food safety—implications for food waste. Curr. Opin. Food Sci..

[23] Madilo F.K., Owusu-Kwarteng J., Parry-Hanson Kunadu A., Tano-Debrah K. (2020). Self-reported use and understanding of food label information among tertiary education students in Ghana. Food Control.

[24] Toma L., Costa Font M., Thompson B. (2020). Impact of consumers’ understanding of date labelling on food waste behaviour. Oper. Res..

[25] Zielińska D., Bilska B., Marciniak-łukasiak K., Łepecka A., Trząskowska M., Neffe-skocińska K., Tomaszewska M., Szydłowska A., Kołożyn-krajewska D. (2020). Consumer understanding of the date of minimum durability of food in association with quality evaluation of food products after expiration. Int. J. Environ. Res. Public Health.

[26] Gašo-Sokač D., Kovač S., Josić D. (2011). Use of proteomic methodology in optimization of processing and quality control of food of animal origin. Food Technol. Biotechnol..

[27] Johnson M.E., Lucey J.A. (2006). Major technological advances and trends in cheese. J. Dairy Sci..

[28] Dambrouck T., Marchal R., Marchal-Delahaut L., Parmentier M., Maujean A., Jeandet P. (2003). Immunodetection of proteins from grapes and yeast in a white wine. J. Agric. Food Chem..

[29] Gagnaire V., Jardin J., Jan G., Lortal S. (2009). Invited review: Proteomics of milk and bacteria used in fermented dairy products: From qualitative to quantitative advances. J. Dairy Sci..

[30] Matissek R., Fischer M. (2021). Lebensmittelanalytik.

[31] Meltretter J., Becker C.M., Pischetsrieder M. (2008). Identification and site-specific relative quantification of β-lactoglobulin modifications in heated milk and dairy products. J. Agric. Food Chem..

[32] Ebner J., Baum F., Pischetsrieder M. (2016). Identification of sixteen peptides reflecting heat and/or storage induced processes by profiling of commercial milk samples. J. Proteom..

[33] Liu Y., Pischetsrieder M. (2017). Identification and Relative Quantification of Bioactive Peptides Sequentially Released during Simulated Gastrointestinal Digestion of Commercial Kefir. J. Agric. Food Chem..

[34] von Oesen T., Treblin M., Clawin-Rädecker I., Martin D., Maul R., Hoffmann W., Schrader K., Wegner B., Bode K., Zink R. (2023). Identification of Marker Peptides for the Whey Protein Quantification in Edam-Type Cheese. Foods.

[35] Cristobal A., Marino F., Post H., Van Den Toorn H.W.P., Mohammed S., Heck A.J.R. (2017). Toward an Optimized Workflow for Middle-Down Proteomics. Anal. Chem..

[36] Giansanti P., Tsiatsiani L., Low T.Y., Heck A.J.R. (2016). Six alternative proteases for mass spectrometry-based proteomics beyond trypsin. Nat. Protoc..

[37] Granato D., de Araújo Calado V.Ô.M., Jarvis B. (2014). Observations on the use of statistical methods in Food Science and Technology. Food Res. Int..

[38] Morschheuser L., Mink K., Horst R., Kallinich C., Rohn S. (2017). Immunological analysis of food proteins using high-performance thin-layer chromatography-immunostaining. J. Chromatogr. A.

[39] Post A.E., Arnold B., Weiss J., Hinrichs J. (2012). Effect of temperature and pH on the solubility of caseins: Environmental influences on the dissociation of α S- and β-casein. J. Dairy Sci..

[40] Pelegrine D.H.G., Gasparetto C.A. (2005). Whey proteins solubility as function of temperature and pH. LWT.

[41] BVL Amtliche Sammlung von Untersuchungsverfahren nach §64 LFGB ASU L00.90-6, 2015.

[42] (2014). DIN Deutsches Institut für Normung e., V. Sensory Analysis-Simple Descriptive Test.

[43] BVL Amtliche Sammlung von Untersuchungsverfahren nach §64 LFGB ASU L00.90-7, 2021.

[44] (2021). DIN Deutsches Institut für Normung e., V. Sensory Analysis-Triangle Test.

[45] Regueiro J., Negreira N., Berntssen M.H.G. (2016). Ion-mobility-derived collision cross section as an additional identification point for multiresidue screening of pesticides in fish feed. Anal. Chem..

[46] Bijlsma L., Berntssen M.H.G., Merel S. (2019). A Refined Nontarget Workflow for the Investigation of Metabolites through the Prioritization by in Silico Prediction Tools. Anal. Chem..

[47] Kim N., Kim K., Choi B.Y., Lee D., Shin Y.S., Bang K.H., Cha S.W., Lee J.W., Choi H.K., Jang D.S. (2011). Metabolomic approach for age discrimination of Panax ginseng using UPLC-Q-Tof MS. J. Agric. Food Chem..

[48] Sumner L.W., Mendes P., Dixon R.A. (2003). Plant metabolomics: Large-scale phytochemistry in the functional genomics era. Phytochemistry.

[49] Röst H.L., Schmitt U., Aebersold R., Malmström L. (2014). pyOpenMS: A Python-based interface to the OpenMS mass-spectrometry algorithm library. Proteomics.

[50] Chavan R.S., Chavan S.R., Khedkar C.D., Jana A.H. (2011). UHT milk processing and effect of plasmin activity on shelf life: A review. Compr. Rev. Food Sci. Food Saf..

[51] Nielsen B.R., Stapelfeldt H., Skibsted L.H. (1997). Early prediction of the shelf-life of medium-heat whole milk powders using stepwise multiple regression and principal component analysis. Int. Dairy J..

[52] Coolbear T., Janin N., Traill R., Shingleton R. (2022). Heat-induced changes in the sensory properties of milk. Int. Dairy J..

[53] Calvo M.M., de la Hoz L. (1992). Flavour of heated milks. A review. Int. Dairy J..

[54] van Boekel M.A.J.S. (1998). Effect of heating on Maillard reactions in milk. Food Chem..

[55] Jo Y., Benoist D.M., Barbano D.M., Drake M.A. (2018). Flavor and flavor chemistry differences among milks processed by high-temperature, short-time pasteurization or ultra-pasteurization. J. Dairy Sci..

[56] Kühn J., Considine T., Singh H. (2006). Interactions of milk proteins and volatile flavor compounds: Implications in the development of protein foods. J. Food Sci..

[57] Hutton J.T., Patton S. (1952). The Origin of Sulfhydryl Groups in Milk Proteins and their Contributions to “Cooked” Flavor. J. Dairy Sci..

[58] Al-Attabi Z., D’Arcy B.R., Deeth H.C. (2009). Volatile sulphur compounds in UHT milk. Crit. Rev. Food Sci. Nutr..

[59] Mehta R.S. (1980). Milk Processed at Ultra-High-Temperatures—A Review. J. Food Prot..

[60] Mahoney R.R. (1998). Galactosyl-oligosaccharide formation during lactose hydrolysis: A review. Food Chem..

[61] Adhikari K., Dooley L.M., Chambers I.V.E., Bhumiratana N. (2010). Sensory characteristics of commercial lactose-free milks manufactured in the United States. LWT.

[62] Aktağ I.G., Hamzalıoğlu A., Gökmen V. (2019). Lactose hydrolysis and protein fortification pose an increased risk for the formation of Maillard reaction products in UHT treated milk products. J. Food Compos. Anal..

[63] Kong Y., Dong Q., Yu Z., Yan H., Liu L., Shen Y. (2023). The effect of lactose and its isomerization product lactulose on functional and structural properties of glycated casein. Food Res. Int..

[64] Montgomery E.M., Hudson C.S. (1930). Relations between rotatory power and structure in the sugar group. XXIX. Synthesis of a new disaccharide ketose (Lactulose) from Lactose. J. Am. Chem. Soc..

[65] Parrish F.W., Talley F.B., Ross K.D., Clark J., Phillips J.G. (1979). Sweetness of Lactulose Relative To Sucrose. J. Food Sci..

[66] Panesar P.S., Kumari S. (2011). Lactulose: Production, purification and potential applications. Biotechnol. Adv..

[67] Scanlan R.A., Lindsay R.C., Libbey L.M., Day E.A. (1968). Heat-Induced Volatile Compounds in Milk. J. Dairy Sci..

[68] Colahan-Sederstrom P.M., Peterson D.G. (2005). Inhibition of key aroma compound generated during ultrahigh-temperature processing of bovine milk via epicatechin addition. J. Agric. Food Chem..

[69] Klockmann S., Reiner E., Bachmann R., Hackl T., Fischer M. (2016). Food Fingerprinting: Metabolomic Approaches for Geographical Origin Discrimination of Hazelnuts (*Corylus avellana*) by UPLC-QTOF-MS. J. Agric. Food Chem..

[70] Bensadek D., Monigatti F., Steen J.A.J., Steen H. (2007). Why b, y’s? Sodiation-induced tryptic peptide-like fragmentation of non-tryptic peptides. Int. J. Mass Spectrom..

[71] Steen H., Mann M. (2004). The ABC’s (and XYZ’s) of peptide sequencing. Nat. Rev. Mol. Cell Biol..

[72] Malinowski J., Klempt M., Clawin-Rädecker I., Lorenzen P.C., Meisel H. (2014). Identification of a NFκB inhibitory peptide from tryptic β-casein hydrolysate. Food Chem..

[73] Tu M., Feng L., Wang Z., Qiao M., Shahidi F., Lu W., Du M. (2017). Sequence analysis and molecular docking of antithrombotic peptides from casein hydrolysate by trypsin digestion. J. Funct. Foods.

[74] Meltretter J., Schmidt A., Humeny A., Becker C.M., Pischetsrieder M. (2008). Analysis of the peptide profile of milk and its changes during thermal treatment and storage. J. Agric. Food Chem..

[75] von Oesen T., Treblin M., Staudacher A., Clawin-Rädecker I., Martin D., Hoffmann W., Schrader K., Bode K., Zink R., Rohn S. (2023). Determination and evaluation of whey protein content in matured cheese via liquid chromatography. LWT.

[76] Van Vlierberghe K., Gavage M., Dieu M., Renard P., Arnould T., Gillard N., Coudijzer K., De Loose M., Gevaert K., Van Poucke C. (2022). Selecting Processing Robust Markers Using High-Resolution Mass Spectrometry for the Detection of Milk in Food Products. J. AOAC Int..

[77] Dalabasmaz S., Ebner J., Pischetsrieder M. (2017). Identification of the Peptide PyroQ-βCasein194-209 as a Highly Specific and Sensitive Marker to Differentiate between Ultrahigh-Temperature Processed (UHT) Milk and Mildly Heated Milk. J. Agric. Food Chem..

