# Supplementary material for: The Shelf Life of Milk—A Novel Concept for the Identification of Marker Peptides Using Multivariate Analysis
Source: Foods. 2024 Mar 8;13(6):831. doi: 10.3390/foods13060831 (PMC10969003; doi:10.3390/foods13060831)
Supplement: Supplementary file 1 [file foods-13-00831-s001.zip › foods-2893908-supplementary.pdf]

foods

Supplementary Materials

The Shelf Life of Milk—A Novel Concept for the Identification of Marker Peptides Using Multivariate Analysis

Lisa-Carina Class [1,2,†], Gesine Kuhnen [1,3,†], Kim Lara Hanisch [1], Svenja Badekow [1], Sascha Rohn [3] and Jürgen Kuballa [1,*]

[1] GALAB Laboratories GmbH, Am Schleusengraben 7, 21029 Hamburg, Germany; lisa-carina.class@galab.de (L.-C.C.); gesine.kuhnen@galab.de (G.K.)
[2] Hamburg School of Food Science, Institute of Food Chemistry, University of Hamburg, Grindelallee 117, 20146 Hamburg, Germany
[3] Department of Food Chemistry and Analysis, Institute of Food Technology and Food Chemistry, Technische Universität Berlin, Gustav-Meyer-Allee 25, 13355, Berlin, Germany; rohn@tu-berlin.de
* Correspondence: juergen.kuballa@galab.de
† Both authors contributed equally to this work.

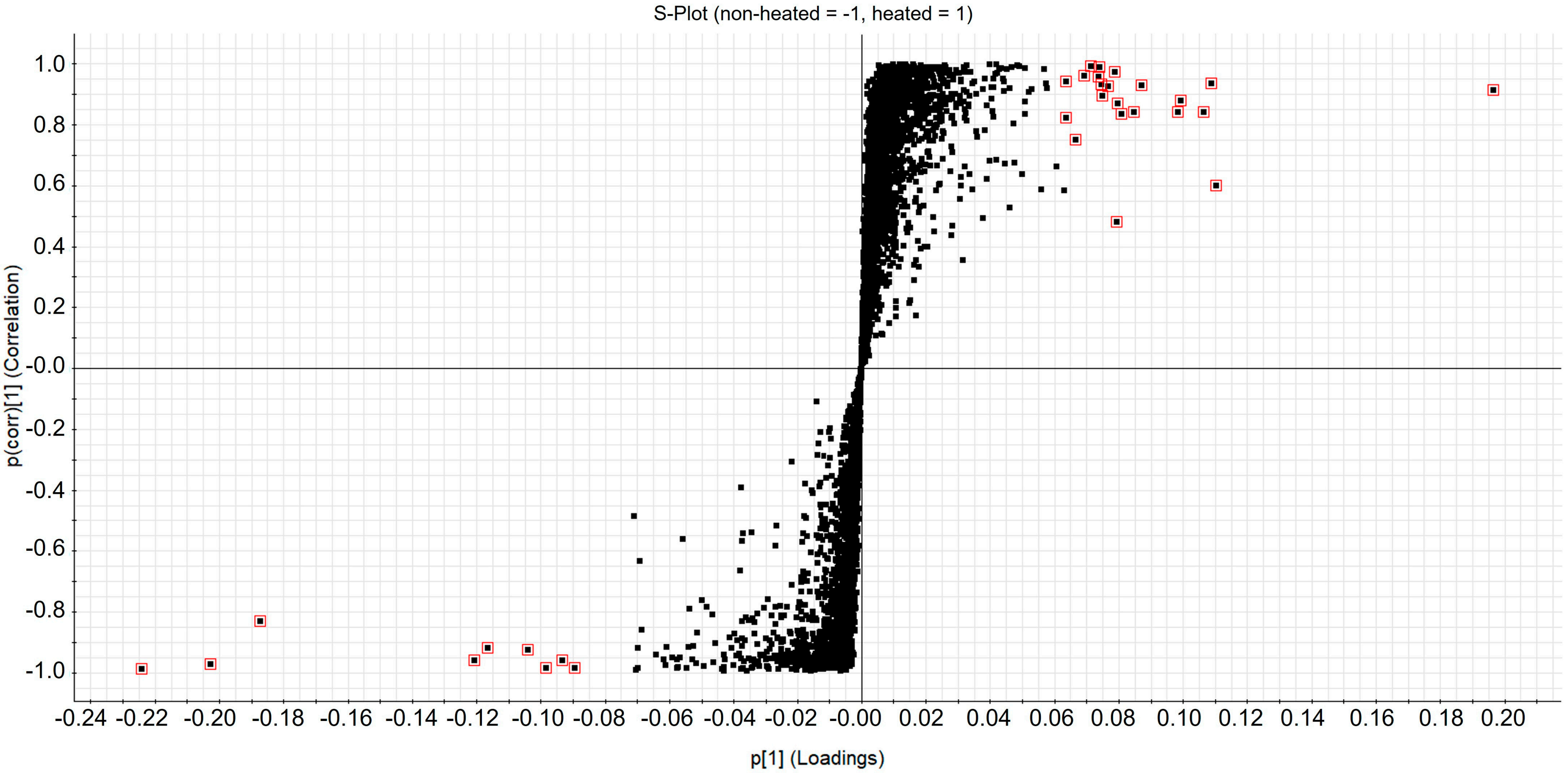

Figure S1. Loading plot (S-plot) of the OPLS-DA to differentiate heated and non-heated milk samples. (non-heated (-1) and heated (1)). The data points in the rectangle marked in red are the points with the greatest variance.

Table S1. Comparison of the tryptic peptide signal intensities obtained from α-Lactalbumin standard after tryptic digestion. Two determinations were carried out for each sample preparation. In one of the sample preparation methods, the standard was used directly for tryptic digestion; in the other method, the standard was heated beforehand. In the table, the occurrence of the tryptic peptide with only one purification method was labelled with a cross (X) in the respective column. The differences in signal intensity were labelled accordingly with an "H" if the signal intensity of the tryptic peptide is higher in the heated samples and with an "NH" if the signal intensity is higher in the unheated sample.

Tryptic Peptide	only appears in the heated samples	only appears in the non-heated samples	shows higher signal intensity in heated (H) or in non-heated (NH)
ELKDLK			H
KILDK	X		
K	X		
L		X	

Table S2. Comparison of the tryptic peptide signal intensities obtained from β-Lactoglobulin standard after tryptic digestion. Two determinations were carried out for each sample preparation. In one of the sample preparation methods, the standard was used directly for tryptic digestion; in the other method, the standard was heated beforehand. In the table, the occurrence of the tryptic peptide with only one purification method was labelled with a cross (X) in the respective column. The differences in signal intensity were labelled accordingly with an "H" if the signal intensity of the tryptic peptide is higher in the heated samples and with an "NH" if the signal intensity is higher in the unheated sample.

Tryptic Peptide	only appears in the heated samples	only appears in the non-heated samples	shows higher signal intensity in heated (H) or in non-heated (NH)
IDALNENK		X	
TPEVDDEALEKFDK			NH
VAGTWYLAMAASDIDLLDAQSAPLRVYYEELKPTPEGDLEILLQK		X	
VLVLDTYKK		X	
VYVEELKPTPEGDLELLQK		X	
FDKALK		X	
TPEVDDEALEKFDK			NH
IIAEK	X		
TPEVDDEALEK	X		
WENGECAQKK	X		

Table S3. Comparison of the tryptic peptide signal intensities obtained from Bovine Serum Albumin (BSA) standard after tryptic digestion. Two determinations were carried out for each sample preparation. In one of the sample preparation methods, the standard was used directly for tryptic digestion; in the other method, the standard was heated beforehand. In the table, the occurrence of the tryptic peptide with only one purification method was labelled with a cross (X) in the respective column. The differences in signal intensity were labelled accordingly with an "H" if the signal intensity of the tryptic peptide is higher in the heated samples and with an "NH" if the signal intensity is higher in the unheated sample.

Tryptic Peptide	only appears in the heated samples	only appears in the non-heated samples	shows higher signal intensity in heated (H) or in non-heated (NH)
LKPDPNTLCDEFK		X	
DVCKNYQEAK	X		
AEFVEVTKLVTDLTK	X		
DAIPENLPPLTADFAEDK	X		
DDSPDLPKLKPDPNTLCDEFK	X		
DTHK	X		
DVCK	X		
FKDLGEEHFK	X		
HKPK	X		
IETMR	X		
KYPQVSTPTLVEVSA	X		
LAK	X		
LVNELTEFAK		X	
RHPEYAVSVLLR	X		
TVMENFVAFVDK	X		
YTR	X		
YTRK	X		

Table S4. Comparison of the tryptic peptide signal intensities obtained from α-s1-Casein standard after tryptic digestion. Two determinations were carried out for each sample preparation. In one of the sample preparation methods, the standard was used directly for tryptic digestion; in the other method, the standard was heated beforehand. In the table, the occurrence of the tryptic peptide with only one purification method was labelled with a cross (X) in the respective column. The differences in signal intensity were labelled accordingly with an "H" if the signal intensity of the tryptic peptide is higher in the heated samples and with an "NH" if the signal intensity is higher in the unheated sample.

Tryptic Peptide	only appears in the heated samples	only appears in the non-heated samples	shows higher signal intensity in heated (H) or in non-heated (NH)
EDVPSERYLGYL EQLLR	X		
EGIHAQQK	X		
EPMIGVNQELA YFYPELFR	X		
FFVAPFPEVFGK	X		
YLGYLEQLLRLK	X		
HIQKEDVPSER	X		
LHSMK	X		
LHSMKEGIHAQ QK	X		

Table S5. Comparison of the tryptic peptide signal intensities obtained from α-s2-Casein standard after tryptic digestion. Two determinations were carried out for each sample preparation. In one of the sample preparation methods, the standard was used directly for tryptic digestion; in the other method, the standard was heated beforehand. In the table, the occurrence of the tryptic peptide with only one purification method was labelled with a cross (X) in the respective column. The differences in signal intensity were labelled accordingly with an "H" if the signal intensity of the tryptic peptide is higher in the heated samples and with an "NH" if the signal intensity is higher in the unheated sample.

Tryptic Peptide	only appears in the heated samples	only appears in the non-heated samples	shows higher signal intensity in heated (H) or in non-heated (NH)
ALNEINQFYQKFPQYLQYLYQGPIVLNPWDQVK	X		
ENLCSTFCKEVVR	X		
FPQYLQYLYQGPIVLNPWDQVK	X		
ISQR	X		
LNFLKK	X		
QEKNMAINPSK	X		
TVDMESTEVFTKK	X		
YQK	X		
YQKFALPQYLK	X		

Table S6. Comparison of the tryptic peptide signal intensities obtained from β-Casein standard after tryptic digestion. Two determinations were carried out for each sample preparation. In one of the sample preparation methods, the standard was used directly for tryptic digestion; in the other method, the standard was heated beforehand. In the table, the occurrence of the tryptic peptide with only one purification method was labelled with a cross (X) in the respective column. The differences in signal intensity were labelled accordingly with an "H" if the signal intensity of the tryptic peptide is higher in the heated samples and with an "NH" if the signal intensity is higher in the unheated sample.

Tryptic Peptide	only appears in the heated samples	only appears in the non-heated samples	shows higher signal intensity in heated (H) or in non-heated (NH)
IEK		X	
IEKFQSEEQQQTEDELQDK		X	
IHPFAQTQSLVYPFPGPIPNSLPQNIPPLTQTPVVVPPFLQPEVMGVSK		X	
KIEK			NH
KIEK		X	
YPVEPFTESQSLTLTDVENLHLPLPLLQSWMHQPLPPTVMFPPQSVLSLSQSKVLPVPQK		X	
ELEELNVPGEIVESLSSSEESITR	X		
EMPFPK	X		
HKEMPFPK	X		
IEK			NH
VKEAMAPK	X		
VLPVPQKAVPYPQR	X		

Table S7. Comparison of the tryptic peptide signal intensities obtained from κ-Casein standard after tryptic digestion. Two determinations were carried out for each sample preparation. In one of the sample preparation methods, the standard was used directly for tryptic digestion; in the other method, the standard was heated beforehand. In the table, the occurrence of the tryptic peptide with only one purification method was labelled with a cross (X) in the respective column. The differences in signal intensity were labelled accordingly with an "H" if the signal intensity of the tryptic peptide is higher in the heated samples and with an "NH" if the signal intensity is higher in the unheated sample.

Tryptic Peptide	only appears in the heated samples	only appears in the non-heated samples	shows higher signal intensity in heated (H) or in non-heated (NH)
AVPYPQRDMOIQAFLLYQEPVLGPVR		X	
EAMAPK		X	
EMPFPK		X	
FQSEEQQQTEDELQDK		X	
GPFPIIV		X	
RELEELNVPGEIVESLSSSEESITR		X	
VKEAMAPK		X	
VLPVPQKAVPYPQR		X	
ELEELNVPGEIVESLSSSEESITR	X		

Table S8. Definition of the Feature names. Summarised are the names of the features in the present research and the assignment of the feature label in Progenesis QI as well as the mass-to-charge ratio (*m/z*) and the retention time.

Feature name	Feature name in Progenesis QI	*m/z*	Retention time [min]
**FT 01**	8.72_655.9955m/z	655.9955	8.72
**FT 02**	8.83_885.4834m/z	885.4834	8.83
**FT 03**	6.55_639.3500m/z	639.3500	6.55
**FT 04**	7.32_635.8622m/z	635.8622	7.32
**FT 05**	9.32_634.3570m/z	634.3570	9.32
**FT 06**	8.91_779.3525n	821.3864	8.91
**FT 07**	8.12_858.4076m/z	858.4076	8.12
**FT 08**	10.72_2066.1443n	697.0494	10.72
**FT 09**	8.22_761.7321m/z	761.7321	8.22
**FT 10**	5.51_379.0258n	443.0417	5.51
**FT 11**	8.38_684.3799m/z	684.3799	8.38
**FT 12**	7.51_412.7191m/z	412.7191	7.51
**FT 13**	5.89_523.2861m/z	523.2861	5.89
**FT 14**	7.62_737.7065m/z	737.7065	7.62
**FT 15**	10.73_1045.0706m/z	1045.0706	10.73
**FT 16**	8.04_791.6306m/z	791.6306	8.04
**FT 17**	9.16_706.3368n	748.3706	9.16
**FT 18**	8.04_791.3799m/z	791.3799	8.04
**FT 19**	9.99_700.4168n	742.4507	9.99
**FT 20**	8.42_1141.0840m/z	1141.0840	8.42
**FT 21**	11.04_2700.5513n	908.5183	11.04
**FT 22**	6.60_412.7530m/z	412.7530	6.60
**FT 23**	6.59_421.7586m/z	421.7586	6.59
**FT 24**	6.11_623.2967m/z	623.2967	6.11
**FT 25**	8.40_2258.1753n	761.0597	8.40
**FT 26**	7.73_880.4767m/z	880.4767	7.73
**FT 27**	10.85_2718.5083n	914.5040	10.85
**FT 28**	7.93_692.4041m/z	692.4041	7.93

Table S9. Comparison of the detected *m/z* from feature FT 02 and the assigned fragments of the tryptic peptide "FPQYLQYLYQGPIVLNPWDQVK". The *m/z* are selected from the low-energy spectra at 8.83 min. The datafile, which was used belongs to a milk sample without heat treatment. The theoretic fragment *m/z* was calculated by a package from *pyOpenMS*. Moreover, the comparison was made with a tolerance of 0.001%.

	observed *m/z*	observed intensities	b-/y-fragment	theoretic *m/z* + mass shift(0.0)/z
**0**	443.723295	255.916107	[y7++]	443.724512
**1**	489.270084	254.137299	[y4+]	489.266742
**2**	500.266952	327.919403	[y8++]	500.266544
**3**	526.759358	84.698685	[b9-H3N1++]	526.758021
**4**	585.300726	1307.098999	[a6-H3N1+]	585.303127
**5**	654.873498	65.088470	[y11++]	654.869166
**6**	683.374994	107.023674	[y12++]	683.379898
**7**	683.380856	167.433807	[y12++]	683.379898
**8**	735.868414	177.000885	[a12++]	735.874448
**9**	747.407178	152.735062	[y13++]	747.409187

	**observed** ***m/z***	**observed intensities**	**b-/y-fragment**	**theoretic** ***m/z*** **+ mass shift(0.0)/z**
**10**	747.416522	705.369873	[y13++]	747.409187
**11**	759.903439	37.373505	[a14-H3N1++]	759.903206
**12**	772.401277	395.942505	[y6+]	772.398819
**13**	828.937973	58.162655	[y14++]	828.940851
**14**	885.481404	13596.849609	[y15++]	885.482884
**15**	886.441193	513.488159	[y7+]	886.441747
**16**	887.459028	243.799072	[b16-H3N1++]	887.464159
**17**	912.464303	39.073715	[a7+]	912.461419
**18**	1018.037324	259.150024	[b17++]	1018.038023

Table S10. Comparison of the detected *m/z* from feature FT 02 and the assigned fragments of the tryptic peptide "FPQYLQYLYQGPIVLNPWDQVK". The *m/z* are selected from the high-energy spectra at 8.83 min. The datafile, which was used belongs to a milk sample without heat treatment. The theoretic fragment *m/z* was calculated by a package from *pyOpenMS*. Moreover, the comparison was made with a tolerance of 0.001%.

	**observed** ***m/z***	**observed intensities**	**b-/y-fragment**	**theoretic** ***m/z*** **+ mass shift(0.0)/z**
**0**	357.213865	404.439026	[y3-H3N1+]	357.213248
**1**	386.703348	327.010651	[y6++]	386.703048
**2**	489.261878	186.056625	[y4+]	489.266742
**3**	489.267589	280.004669	[y4+]	489.266742
**4**	675.348262	128.366196	[y5+]	675.346055
**5**	747.402546	328.565063	[y13++]	747.409187
**6**	747.409497	283.877625	[y13++]	747.409187
**7**	754.391219	57.468731	[y6-H2O1+]	754.388254
**8**	772.399998	1682.897095	[y6+]	772.398819
**9**	806.413784	47.222878	[b13++]	806.413938
**10**	885.477971	4634.312012	[y15++]	885.482884
**11**	885.491166	847.708313	[y15++]	885.482884
**12**	886.433736	432.618530	[y7+]	886.441747
**13**	886.447706	508.166779	[y7+]	886.441747
**14**	887.459257	112.064186	[b16-H3N1++]	887.464159
**15**	999.523037	523.862244	[y8+]	999.525812
**16**	1018.045055	84.793427	[b17++]	1018.038023
**17**	1098.599986	167.001526	[y9+]	1098.594226
**18**	1308.729537	295.342102	[y11+]	1308.731055
**19**	1348.724393	47.540222	[y12-H3N1+]	1348.725970
**20**	1365.756221	333.451599	[y12+]	1365.752519

Table S11. Comparison of the detected *m*/*z* from feature FT 03 and the assigned isotopes of the tryptic peptide "VLPVPQKAVPYPQR" modified with lactulosyllysine. The *m*/*z* are selected from the low-energy spectra at 6.55 min. The datafile, which was used belongs to a milk sample without heat treatment. The theoretic fragment *m*/*z* was calculated by a package from *pyOpenMS*. Moreover, the comparison was made with a tolerance of 0.001%. *m*/*z* 639.6857: 31.4965%; *m*/*z* 640.0193: 1.9260%; *m*/*z* 640.3538: 1.8123% are those isotopes, which were not found in the low-energy spectra.

	observed *m*/*z*	observed intensity	isotope *m*/*z* (charge: 3)	natural isotope abundance [%]
**0**	639.349547	2799.458496	639.351243	33.472475
**1**	639.681844	2168.276367	639.683588	2.472674
**2**	640.018127	1193.042480	640.018040	2.326708
**3**	640.023333	1118.282471	640.020147	14.648296
**4**	640.349521	467.648651	640.352492	1.082097
**5**	640.354814	391.677673	640.354598	4.488907
**6**	640.687318	277.081116	640.688231	0.842860
**7**	640.692947	158.940826	640.689050	1.019567

Table S12. Comparison of the detected *m*/*z* from feature FT 03 and the assigned fragments of the tryptic peptide "VLPVPQKAVPYPQR" modified with lactulosyllysine. The *m*/*z* are selected from the low-energy spectra at 6.55 min. The datafile, which was used belongs to a milk sample without heat treatment. The theoretic fragment *m*/*z* was calculated by a package from *pyOpenMS*. Moreover, the comparison was made with a tolerance of 0.001%.

	observed *m*/*z*	observed intensities	b-/y-fragment	theoretic *m*/*z* + mass shift(324.105650638)/z
**0**	509.273931	96.018341	[a2+]	509.270491
**1**	569.272633	42.263874	[y7-H3N1++]	569.269148
**2**	628.846919	113.068466	[b9++]	628.852848
**3**	633.310624	90.548813	[y8-H3N1++]	633.316629
**4**	633.320980	162.190643	[y8-H3N1++]	633.316629
**5**	677.377094	56.607109	[b10++]	677.379230
**6**	754.389933	30.638016	[y10++]	754.385575
**7**	852.450188	96.651726	[y12++]	852.446164

Table S13. Comparison of the detected *m*/*z* from feature FT 03 and the assigned fragments of the tryptic peptide "VLPVPQKAVPYPQR" modified with lactulosyllysine. The *m*/*z* are selected from the high-energy spectra at 6.55 min. The datafile, which was used belongs to a milk sample without heat treatment. The theoretic fragment *m*/*z* was calculated by a package from *pyOpenMS*. Moreover, the comparison was made with a tolerance of 0.001%.

	observed *m*/*z*	observed intensities	b-/y-fragment	theoretic *m*/*z* + mass shift(324.105650638)/z
**0**	520.256373	131.190186	[y6-C1H2N1O1++]	520.257046
**1**	744.909913	155.216385	[a11++]	744.913437
**2**	744.916580	143.374603	[a11++]	744.913437

Table S14. Comparison of the detected *m*/*z* from feature FT 04 and the assigned fragments of the tryptic peptide "DMPIQAFLLYQEPVLGPVR". The *m*/*z* are selected from the low-energy spectra at 7.32 min. The datafile, which was used belongs to a milk sample without heat treatment. The theoretic fragment *m*/*z* was calculated by a package from *pyOpenMS*. Moreover, the comparison was made with a tolerance of 0.001%.

	**observed *m*/*z***	**observed intensities**	**b-/y-fragment**	**theoretic *m*/*z* + mass shift(0.0)/z**
**0**	186.123648	164.566803	[y3++]	186.123704
**1**	271.176806	390.390137	[y5++]	271.176468
**2**	371.238210	420.431274	[y3+]	371.240131
**3**	371.242029	460.522614	[y3+]	371.240131
**4**	428.261638	1368.447632	[y4+]	428.261596
**5**	497.787510	216.782837	[y9++]	497.787644
**6**	524.255974	84.367149	[b6-H3N1+]	524.253733
**7**	541.346574	933.037720	[y5+]	541.345660
**8**	579.316089	105.669289	[y10++]	579.319308
**9**	579.324544	140.960388	[y10++]	579.319308
**10**	627.347070	76.502747	[y11-H3N1++]	627.348066
**11**	635.863041	96284.414062	[y11++]	635.861340
**12**	646.834829	312.362946	[a11++]	646.839019
**13**	658.840055	136.312119	[b12-H2O1++]	658.839019
**14**	737.465888	327.087860	[y7+]	737.466838
**15**	775.374668	119.014076	[a7+]	775.380726
**16**	843.480763	90.001213	[y15-C1H2N1O1++]	843.478606
**17**	994.564882	97.023308	[y9+]	994.568011
**18**	994.573013	82.031235	[y9+]	994.568011

Table S15. Comparison of the detected *m*/*z* from feature FT 04 and the assigned fragments of the tryptic peptide "DMPIQAFLLYQEPVLGPVR". The *m*/*z* are selected from the high-energy spectra at 7.32 min. The datafile, which was used belongs to a milk sample without heat treatment. The theoretic fragment *m*/*z* was calculated by a package from *pyOpenMS*. Moreover, the comparison was made with a tolerance of 0.001%.

	**observed *m*/*z***	**observed intensities**	**b-/y-fragment**	**theoretic *m*/*z* + mass shift(0.0)/z**
**0**	354.213900	218.461349	[y3-H3N1+]	354.213582
**1**	411.236014	195.002670	[y4-H3N1+]	411.235046
**2**	428.259688	1448.968018	[y4+]	428.261596
**3**	488.782268	785.101929	[y9-H2O1++]	488.782361
**4**	489.278093	384.804199	[y9-H3N1++]	489.274369
**5**	497.789963	540.090698	[y9++]	497.787644
**6**	541.347541	1075.765747	[y5+]	541.345660
**7**	635.863746	2114.915771	[y11++]	635.861340
**8**	640.407846	171.217316	[y6+]	640.414074
**9**	640.419867	276.245911	[y6+]	640.414074
**10**	737.467593	7326.155273	[y7+]	737.466838
**11**	844.474910	200.025970	[y15-C1H2N2++]	844.474527
**12**	848.501736	157.007874	[y8-H2O1+]	848.498868
**13**	849.483126	82.177948	[y8-H3N1+]	849.482884
**14**	866.508049	1422.489380	[y8+]	866.509433
**15**	868.513693	216.123566	[a9-H2O1+]	868.511345
**16**	976.554107	109.813156	[y9-H2O1+]	976.557446

	observed *m/z*	observed intensities	b-/y-fragment	theoretic *m/z* + mass shift(0.0)/z
**17**	976.566866	96.624908	[y9-H2O1+]	976.557446
**18**	994.576729	1088.219849	[y9+]	994.568011
**19**	1140.608884	97.000038	[y10-H3N1+]	1140.604791
**20**	1157.620977	60.000103	[y10+]	1157.631340
**21**	1157.629286	82.797470	[y10+]	1157.631340
**22**	1159.633131	60.177803	[a11-H2O1+]	1159.633253
**23**	1159.641436	30.056572	[a11-H2O1+]	1159.633253
**24**	1270.716098	97.032471	[y11+]	1270.715404
**25**	1270.725425	89.139221	[y11+]	1270.715404

Table S16. Comparison of the detected *m/z* from feature FT 05 and the assigned isotopes of the tryptic peptide "YLGYLEQLLR". The *m/z* are selected from the low-energy spectra at 9.32 min. The datafile, which was used belongs to a milk sample without heat treatment. The theoretic fragment *m/z* was calculated by a package from *pyOpenMS*. Moreover, the comparison was made with a tolerance of 0.001%. *m/z* 639.6857: 31.4965%; *m/z* 634.8550: 2.4499%; *m/z* 635.3566: 1.5898%; *m/z* 635.3586: 1.5577% are those isotopes, which were not found in the low-energy spectra.

	observed *m/z*	observed intensity	isotope *m/z* (charge: 2)	natural isotope abundance [%]
**0**	634.357631	7225.833008	634.356440	47.376698
**1**	634.857909	4149.336426	634.858117	30.744812
**2**	635.359816	1779.754150	635.359795	9.809563
**3**	635.863418	251.009232	635.861472	2.051220

Table S17. Comparison of the detected *m/z* from feature FT 05 and the assigned fragments of the tryptic peptide "YLGYLEQLLR". The *m/z* are selected from the low-energy spectra at 9.32 min. The datafile, which was used belongs to a milk sample without heat treatment. The theoretic fragment *m/z* was calculated by a package from *pyOpenMS*. Moreover, the comparison was made with a tolerance of 0.001%.

	observed *m/z*	observed intensities	b-/y-fragment	theoretic *m/z* + mass shift(0.0)/z
**0**	277.152532	176.044022	[b2+]	277.154670
**1**	277.156800	355.495544	[b2+]	277.154670
**2**	288.203302	776.423584	[y2+]	288.203017
**3**	370.187557	448.236084	[b6++]	370.186699
**4**	386.238765	46.639008	[y6++]	386.239797
**5**	401.284280	900.623840	[y3+]	401.287082
**6**	401.288078	758.442261	[y3+]	401.287082
**7**	469.242912	202.263611	[a4+]	469.244548
**8**	496.282959	180.196686	[y8++]	496.282194
**9**	497.242906	885.471680	[b4+]	497.239463
**10**	512.316407	102.027161	[y4-H3N1+]	512.319111
**11**	529.342324	796.221741	[y4+]	529.345660
**12**	529.350684	773.538818	[y4+]	529.345660
**13**	552.819048	112.809845	[y9++]	552.824226
**14**	582.330634	161.839737	[a5+]	582.328613
**15**	641.360781	101.014984	[y5-H3N1+]	641.361705
**16**	658.387864	415.424500	[y5+]	658.388254
**17**	771.471907	578.683838	[y6+]	771.472318
**18**	867.416992	28.760794	[b7+]	867.424700

	observed *m*/*z*	observed intensities	b-/y-fragment	theoretic *m*/*z* + mass shift(0.0)/z
**19**	867.428313	153.467194	[b7+]	867.424700
**20**	991.552332	889.014038	[y8+]	991.557112
**21**	1093.583334	112.186592	[b9+]	1093.592829
**22**	1093.594996	76.341522	[b9+]	1093.592829
**23**	1104.637195	84.696510	[y9+]	1104.641176
**24**	1104.649987	84.158485	[y9+]	1104.641176

Table S18. Comparison of the detected *m*/*z* from feature FT 05 and the assigned fragments of the tryptic peptide "YLGYLEQLLR". The *m*/*z* are selected from the high-energy spectra at 9.32 min. The datafile, which was used belongs to a milk sample without heat treatment. The theoretic fragment *m*/*z* was calculated by a package from *pyOpenMS*. Moreover, the comparison was made with a tolerance of 0.001%.

	observed *m*/*z*	observed intensities	b-/y-fragment	theoretic *m*/*z* + mass shift(0.0)/z
**0**	249.160784	4819.333984	[a2+]	249.159755
**1**	271.177181	121.434151	[y2-H3N1+]	271.176468
**2**	277.154683	887.438110	[b2+]	277.154670
**3**	288.201407	567.023560	[y2+]	288.203017
**4**	334.175034	840.761475	[b3+]	334.176134
**5**	401.286357	626.057617	[y3+]	401.287082
**6**	469.243860	844.263428	[a4+]	469.244548
**7**	497.240140	326.000122	[b4+]	497.239463
**8**	512.321068	453.182953	[y4-H3N1+]	512.319111
**9**	529.347343	2129.488770	[y4+]	529.345660
**10**	552.826858	215.488342	[y9++]	552.824226
**11**	582.332624	245.180695	[a5+]	582.328613
**12**	610.325336	244.490616	[b5+]	610.323528
**13**	640.375135	540.342102	[y5-H2O1+]	640.377689
**14**	658.359400	160.699646	[a7-H2O1+]	658.355891
**15**	658.390529	4175.032715	[y5+]	658.388254
**16**	711.372074	84.307175	[a6+]	711.371207
**17**	771.436529	178.000565	[a8-H2O1+]	771.439955
**18**	771.471747	4619.915527	[y6+]	771.472318
**19**	772.428858	167.132935	[a8-H3N1+]	772.423971
**20**	934.539231	1323.020264	[y7+]	934.535648
**21**	974.522840	65.450111	[y8-H3N1+]	974.530563
**22**	974.534269	149.011642	[y8-H3N1+]	974.530563
**23**	980.504809	349.932098	[b8+]	980.508765
**24**	991.556803	18977.550781	[y8+]	991.557112
**25**	1093.582574	112.944908	[b9+]	1093.592829
**26**	1104.642191	429.891022	[y9+]	1104.641176
**27**	1104.649774	513.832947	[y9+]	1104.641176

Table S19. Comparison of the detected *m*/*z* from feature FT 12 the assigned fragments of the tryptic peptide "EPMIGVNQELAYFYPELFR". The *m*/*z* are selected from the low-energy spectra at 7.51 min. The datafile, which was used belongs to a milk sample without heat treatment. The theoretic fragment *m*/*z* was calculated by a package from *pyOpenMS*. Moreover, the comparison was made with a tolerance of 0.001%.

	observed *m*/*z*	observed intensities	b-/y-fragment	theoretic *m*/*z* + mass shift(0.0)/z
**0**	175.117887	167.069748	[y1+]	175.118953
**1**	322.185234	428.743927	[y2+]	322.187367
**2**	331.186756	14582.126953	[y5++]	331.187033
**3**	404.207220	1725.908325	[y6-H3N1++]	404.205423
**4**	412.719144	221004.312500	[y6++]	412.718698
**5**	522.295780	183.148911	[y4-C1H2N2+]	522.292228
**6**	546.298119	75.509941	[y4-H2O1+]	546.303461
**7**	564.310062	3686.668945	[y4+]	564.314026
**8**	564.315955	4162.120605	[y4+]	564.314026
**9**	566.313550	235.691055	[a7-H2O1+]	566.311917
**10**	661.363452	3682.815918	[y5+]	661.366790
**11**	661.369891	4628.595215	[y5+]	661.366790
**12**	713.370411	65.314041	[a7+]	713.365076
**13**	824.393126	113.910828	[a9-H3N1+]	824.397105
**14**	824.431572	1268.237061	[y6+]	824.430119

Table S20. Comparison of the detected *m*/*z* from feature FT 12 and the assigned fragments of the tryptic peptide "EPMIGVNQELAYFYPELFR". The *m*/*z* are selected from the high-energy spectra at 7.51 min. The datafile, which was used belongs to a milk sample without heat treatment. The theoretic fragment *m*/*z* was calculated by a package from *pyOpenMS*. Moreover, the comparison was made with a tolerance of 0.001%.

	observed *m*/*z*	observed intensities	b-/y-fragment	theoretic *m*/*z* + mass shift(0.0)/z
**0**	158.092206	167.000397	[y1-H3N1+]	158.092404
**1**	175.120225	438.959412	[y1+]	175.118953
**2**	199.106906	179.460785	[a2+]	199.107720
**3**	227.101518	337.961761	[b2+]	227.102635
**4**	322.190168	510.229614	[y2+]	322.187367
**5**	331.185180	409.672607	[y5++]	331.187033
**6**	331.188783	899.738586	[y5++]	331.187033
**7**	418.242650	75.316315	[y3-H3N1+]	418.244882
**8**	435.271410	1865.502686	[y3+]	435.271432
**9**	546.303803	279.010437	[y4-H2O1+]	546.303461
**10**	564.314712	6749.700195	[y4+]	564.314026
**11**	566.313181	235.528442	[a7-H2O1+]	566.311917
**12**	661.363874	4248.802734	[y5+]	661.366790
**13**	661.369258	3866.999268	[y5+]	661.366790
**14**	824.398111	70.179008	[a9-H3N1+]	824.397105
**15**	824.432270	1579.476074	[y6+]	824.430119

Table S21. Comparison of the detected *m*/*z* from feature FT 24 and the assigned isotopes of the tryptic peptide "TPEVDDEALEK". The *m*/*z* are selected from the low-energy spectra at 6.11 min. The datafile, which was used belongs to a milk sample without heat treatment. The theoretic fragment *m*/*z* was calculated by a package from *pyOpenMS*. Moreover, the comparison was made with a tolerance of 0.001%. *m*/*z* 623.7950: 2.2692%; *m*/*z* 624.2986: 2.4198%; *m*/*z* 624.8002: 1.3610% are those isotopes, which were not found in the low-energy spectra.

	**observed *m*/*z***	**observed intensity**	**isotope *m*/*z* (charge: 2)**	**natural isotope abundance [%]**
**0**	623.296911	108235.835938	623.296443	51.197165
**1**	623.798246	58462.007812	623.798120	28.794202
**2**	624.299822	17564.218750	624.299798	7.941473
**3**	624.801254	4049.162109	624.801475	1.431547

Table S22. Comparison of the detected *m*/*z* from feature FT 24 and the assigned fragments of the tryptic peptide "TPEVDDEALEK". The *m*/*z* are selected from the low-energy spectra at 6.11 min. The datafile, which was used belongs to a milk sample without heat treatment. The theoretic fragment *m*/*z* was calculated by a package from *pyOpenMS*. Moreover, the comparison was made with a tolerance of 0.001%.

	**observed *m*/*z***	**observed intensities**	**b-/y-fragment**	**theoretic *m*/*z* + mass shift(0.0)/z**
**0**	195.124949	66.123116	[y3++]	195.123370
**1**	258.145247	97.156906	[y2-H2O1+]	258.144834
**2**	372.213787	170.572998	[y3-H3N1+]	372.212914
**3**	395.190828	84.022385	[a5-H2O1+]	395.192513
**4**	460.276482	109.444069	[y4+]	460.276578
**5**	589.320891	84.069595	[y5+]	589.319172
**6**	819.368688	92.591141	[y7+]	819.373060

Table S23. Comparison of the detected *m*/*z* from feature FT 24 and the assigned fragments of the tryptic peptide "TPEVDDEALEK". The *m*/*z* are selected from the high-energy spectra at 6.11 min. The datafile, which was used belongs to a milk sample without heat treatment. The theoretic fragment *m*/*z* was calculated by a package from *pyOpenMS*. Moreover, the comparison was made with a tolerance of 0.001%.

	**observed *m*/*z***	**observed intensities**	**b-/y-fragment**	**theoretic *m*/*z* + mass shift(0.0)/z**
**0**	199.106459	375.028381	[b2+]	199.107720
**1**	258.145480	204.021255	[y2-H2O1+]	258.144834
**2**	276.155759	138.618027	[y2+]	276.155399
**3**	328.148808	291.076233	[b3+]	328.150314
**4**	372.213754	113.816612	[y3-H3N1+]	372.212914
**5**	389.241785	265.017670	[y3+]	389.239463
**6**	427.219851	204.881119	[b4+]	427.218728
**7**	460.274338	406.961517	[y4+]	460.276578
**8**	460.279481	311.681641	[y4+]	460.276578
**9**	510.221179	108.387672	[a6-H2O1+]	510.219457
**10**	542.249799	89.100632	[b5+]	542.245673
**11**	571.306452	108.830711	[y5-H2O1+]	571.308607
**12**	572.769568	840.795715	[y10++]	572.772055
**13**	572.774228	1422.267944	[y10++]	572.772055
**14**	589.314673	127.318443	[y5+]	589.319172
**15**	639.266441	24.990620	[a7-H2O1+]	639.262052
**16**	657.266944	83.318214	[b6+]	657.272617

	**observed *m*/*z***	**observed intensities**	**b-/y-fragment**	**theoretic *m*/*z* + mass shift(0.0)/z**
**17**	786.317817	56.142975	[b7+]	786.315211
**18**	801.367887	18.065153	[y7-H2O1+]	801.362495
**19**	819.368205	422.318085	[y7+]	819.373060
**20**	819.377352	1402.708496	[y7+]	819.373060
**21**	918.436692	968.250427	[y8+]	918.441474
**22**	1029.475284	159.722168	[y9-H2O1+]	1029.473504
**23**	1030.465759	66.010056	[y9-H3N1+]	1030.457520
**24**	1047.485854	522.755249	[y9+]	1047.484069
**25**	1099.480814	78.054085	[b10+]	1099.478984
**26**	1144.544689	137.001205	[y10+]	1144.536833

Table S24. Comparison of the detected *m*/*z* from feature FT 26 and the assigned isotopes of the tryptic peptide "HQGLPQEVLNENLLR". The *m*/*z* are selected from the low-energy spectra at 7.73 min. The datafile, which was used belongs to a milk sample without heat treatment. The theoretic fragment *m*/*z* was calculated by a package from *py-OpenMS*. Moreover, the comparison was made with a tolerance of 0.001%. *m*/*z* 623.7950: 2.2692%; *m*/*z* 624.2986: 2.4198%; *m*/*z* 624.8002: 1.3610% are those isotopes, which were not found in the low-energy spectra.

	**observed *m*/*z***	**observed intensity**	**isotope *m*/*z* (charge: 2)**	**natural isotope abundance [%]**
**0**	880.474710	24790.697266	880.476670	37.562159
**1**	880.977306	26732.843750	880.978348	30.875921
**2**	881.472083	5088.869141	881.476865	2.737034
**3**	881.479062	12157.312500	881.478797	1.852560
**4**	881.970000	2040.455444	881.978543	1.110113
**5**	881.983850	2514.644775	881.981703	3.340975

Table S25. Comparison of the detected *m*/*z* from feature FT 26 and the assigned fragments of the tryptic peptide "HQGLPQEVLNENLLR". The *m*/*z* are selected from the low-energy spectra at 7.73 min. The datafile, which was used belongs to a milk sample without heat treatment. The theoretic fragment *m*/*z* was calculated by a package from *py-OpenMS*. Moreover, the comparison was made with a tolerance of 0.001%.

	**observed *m*/*z***	**observed intensities**	**b-/y-fragment**	**theoretic *m*/*z* + mass shift(0.0)/z**
**0**	288.203454	2166.562500	[y2+]	288.203017
**1**	323.144152	583.153076	[b3+]	323.146231
**2**	331.175000	365.575104	[b6++]	331.174457
**3**	395.697485	1410.541504	[b7++]	395.695754
**4**	431.234105	437.978912	[a8++]	431.232504
**5**	436.229876	1258.516113	[b4+]	436.230295
**6**	436.250493	861.743530	[y7++]	436.253437
**7**	445.230714	6836.215332	[b8++]	445.229961
**8**	487.770874	112.722786	[a9++]	487.774536
**9**	501.773972	11109.219727	[b9++]	501.771994
**10**	515.328829	1376.638062	[y4+]	515.330010
**11**	533.279785	436.000000	[b5+]	533.283060
**12**	541.306167	145.464584	[y9-H2O1++]	541.303658
**13**	558.796505	2022.006348	[b10++]	558.793458
**14**	607.316673	146.416977	[a7-H2O1+]	607.319840
**15**	608.306307	151.034225	[a7-H3N1+]	608.303856

	observed *m*/*z*	observed intensities	b-/y-fragment	theoretic *m*/*z* + mass shift(0.0)/z
**16**	623.315740	3406.808350	[b11++]	623.314755
**17**	644.370124	690.959167	[y5+]	644.372604
**18**	661.339954	1114.694580	[b6+]	661.341638
**19**	662.863732	185.022385	[y11++]	662.864612
**20**	680.336423	311.311737	[b12++]	680.336219
**21**	722.882824	276.033875	[a13++]	722.880794
**22**	736.877525	3347.774658	[b13++]	736.878251
**23**	736.884930	2634.708984	[b13++]	736.878251
**24**	740.406412	127.003098	[y6-H2O1+]	740.404967
**25**	758.408279	2212.605713	[y6+]	758.415532
**26**	758.422382	4152.317383	[y6+]	758.415532
**27**	790.383726	1493.919678	[b7+]	790.384232
**28**	793.423953	3004.015381	[b14++]	793.420283
**29**	854.476413	329.001099	[y7-H3N1+]	854.473047
**30**	871.501047	1838.831055	[y7+]	871.499597
**31**	889.449961	874.387634	[b8+]	889.452646
**32**	889.460174	1004.910095	[b8+]	889.452646
**33**	970.560664	426.653412	[y8+]	970.568011
**34**	1002.539375	185.065292	[b9+]	1002.536711
**35**	1002.546545	236.008301	[b9+]	1002.536711
**36**	1082.579798	115.192177	[y9-H3N1+]	1082.584056

Table S26. Comparison of the detected *m*/*z* from feature FT 26 and the assigned fragments of the tryptic peptide "HQGLPQEVLNENLLR". The *m*/*z* are selected from the high-energy spectra at 7.73 min. The datafile, which was used belongs to a milk sample without heat treatment. The theoretic fragment *m*/*z* was calculated by a package from *py-OpenMS*. Moreover, the comparison was made with a tolerance of 0.001%.

	observed *m*/*z*	observed intensities	b-/y-fragment	theoretic *m*/*z* + mass shift(0.0)/z
**0**	175.119943	883.940674	[y1+]	175.118953
**1**	266.124387	803.127869	[b2+]	266.124767
**2**	288.203498	653.244568	[y2+]	288.203017
**3**	304.165729	255.122787	[a7-H2O1++]	304.163558
**4**	323.148619	983.171265	[b3+]	323.146231
**5**	381.696102	305.447754	[a7++]	381.698297
**6**	395.699009	381.910095	[b7++]	395.695754
**7**	401.289186	454.585144	[y3+]	401.287082
**8**	408.236984	2049.832764	[a4+]	408.235380
**9**	436.227398	4592.930176	[b4+]	436.230295
**10**	436.231596	5037.363770	[b4+]	436.230295
**11**	445.233734	661.727966	[b8++]	445.229961
**12**	498.300854	528.005066	[y4-H3N1+]	498.303461
**13**	550.307554	385.885406	[y9++]	550.308941
**14**	623.309665	182.591934	[b11++]	623.314755
**15**	626.365766	368.077087	[y5-H2O1+]	626.362039
**16**	627.342139	196.636047	[y5-H3N1+]	627.346055
**17**	644.366532	1405.881104	[y5+]	644.372604
**18**	654.349463	218.214401	[y11-H3N1++]	654.351337

	observed *m/z*	observed intensities	b-/y-fragment	theoretic *m/z* + mass shift(0.0)/z
**19**	661.345874	1652.000732	[b6+]	661.341638
**20**	662.864011	276.739227	[y11++]	662.864612
**21**	741.382980	436.078278	[y6-H3N1+]	741.388983
**22**	741.391028	785.444885	[y6-H3N1+]	741.388983
**23**	758.408039	2488.602539	[y6+]	758.415532
**24**	758.416336	5939.995117	[y6+]	758.415532
**25**	790.390327	2895.968994	[b7+]	790.384232
**26**	793.426252	168.972794	[b14++]	793.420283
**27**	811.953633	381.072235	[y14++]	811.946665
**28**	854.466248	781.160645	[y7-H3N1+]	854.473047
**29**	854.474217	659.851013	[y7-H3N1+]	854.473047
**30**	861.458389	278.655762	[a8+]	861.457731
**31**	871.505779	4911.960449	[y7+]	871.499597
**32**	889.455657	2478.580078	[b8+]	889.452646
**33**	970.570919	1125.309937	[y8+]	970.568011
**34**	1002.542933	72.925217	[b9+]	1002.536711
**35**	1081.606820	91.156273	[y9-H2O1+]	1081.600040
**36**	1082.589471	91.311462	[y9-H3N1+]	1082.584056
**37**	1099.615683	1000.241272	[y9+]	1099.610605
**38**	1324.708830	794.559021	[y11+]	1324.721948
**39**	1324.731284	910.690857	[y11+]	1324.721948
**40**	1494.827592	511.720215	[y13+]	1494.827476
**41**	1494.838604	295.452911	[y13+]	1494.827476

Table S27. Comparison of the detected *m/z* from feature FT 28 and the assigned fragments of the tryptic peptide "DMPIQAFLLYQEPVLGPVR". The *m/z* are selected from the low-energy spectra at 7.93 min. The datafile, which was used belongs to a milk sample without heat treatment. The theoretic fragment *m/z* was calculated by a package from *pyOpenMS*. Moreover, the comparison was made with a tolerance of 0.001%.

	observed *m/z*	observed intensities	b-/y-fragment	theoretic *m/z* + mass shift(0.0)/z
**0**	186.123408	505.894745	[y3++]	186.123704
**1**	214.633414	417.668091	[y4++]	214.634436
**2**	271.177194	1736.361084	[y5++]	271.176468
**3**	369.235302	574.293091	[y7++]	369.237057
**4**	371.240442	477.215363	[y3+]	371.240131
**5**	428.260739	2231.962646	[y4+]	428.261596
**6**	541.341947	2013.457275	[y5+]	541.345660
**7**	541.350312	1037.490356	[y5+]	541.345660
**8**	585.266250	696.656067	[b5+]	585.270112
**9**	692.403171	179430.218750	[y12++]	692.403373
**10**	693.372575	510.424316	[a13-H2O1++]	693.367944
**11**	693.861554	218.234055	[a13-H3N1++]	693.859952
**12**	775.374768	382.344940	[a7+]	775.380726
**13**	843.480208	674.415039	[y15-C1H2N1O1++]	843.478606
**14**	866.513413	396.228912	[y8+]	866.509433
**15**	994.564963	145.004761	[y9+]	994.568011

Table S28. Comparison of the detected *m*/*z* from feature FT 28 and the assigned fragments of the tryptic peptide "DMPIQAFLLYQEPVLGPVR". The *m*/*z* are selected from the high-energy spectra at 7.93 min. The datafile, which was used belongs to a milk sample without heat treatment. The theoretic fragment *m*/*z* was calculated by a package from *pyOpenMS*. Moreover, the comparison was made with a tolerance of 0.001%.

	observed *m*/*z*	observed intensities	b-/y-fragment	theoretic *m*/*z* + mass shift(0.0)/z
**0**	293.135913	274.114288	[b5++]	293.138694
**1**	354.211483	408.692902	[y3-H3N1+]	354.213582
**2**	354.216577	292.803070	[y3-H3N1+]	354.213582
**3**	371.240348	512.344238	[y3+]	371.240131
**4**	411.235754	450.250671	[y4-H3N1+]	411.235046
**5**	428.264290	4763.102539	[y4+]	428.261596
**6**	488.778644	366.518524	[y9-H2O1++]	488.782361
**7**	541.344647	1509.797607	[y5+]	541.345660
**8**	541.350214	2535.115234	[y5+]	541.345660
**9**	570.805200	549.311646	[y10-H3N1++]	570.806034
**10**	579.319241	3754.706055	[y10++]	579.319308
**11**	580.319532	512.248474	[a11-H2O1++]	580.320265
**12**	635.863096	569.918274	[y11++]	635.861340
**13**	640.413677	236.266098	[y6+]	640.414074
**14**	640.420324	165.128204	[y6+]	640.414074
**15**	683.397378	73.167892	[y12-H2O1++]	683.398090
**16**	683.884620	296.969482	[y12-H3N1++]	683.890098
**17**	692.405265	14596.930664	[y12++]	692.403373
**18**	720.439574	400.729126	[y7-H3N1+]	720.440289
**19**	737.465319	22300.167969	[y7+]	737.466838
**20**	844.466764	255.014053	[y15-C1H2N2++]	844.474527
**21**	848.498391	547.676086	[y8-H2O1+]	848.498868
**22**	866.511478	4218.267090	[y8+]	866.509433
**23**	868.513581	363.016144	[a9-H2O1+]	868.511345
**24**	976.562116	329.098999	[y9-H2O1+]	976.557446
**25**	977.540598	818.003540	[y9-H3N1+]	977.541462
**26**	1157.628680	2189.772217	[y10+]	1157.631340
**27**	1159.637960	760.468750	[a11-H2O1+]	1159.633253
**28**	1160.618125	157.829926	[a11-H3N1+]	1160.617269
**29**	1383.800133	223.866699	[y12+]	1383.799469
